# Supplementary material for: Meta-analysis of gut microbiome studies identifies disease-specific and shared responses
Source: Nat Commun. 2017 Dec 5;8:1784. doi: 10.1038/s41467-017-01973-8 (PMC5716994; doi:10.1038/s41467-017-01973-8)
Supplement: Supplementary file 2 — Supplementary Information [file 41467_2017_1973_MOESM2_ESM.pdf]

Supplementary Information

Supplementary Note 1: Re-processing and re-analyzing raw data yields results which are generally consistent with previously published results

Our re-analyses of the 29 studies were largely consistent with the originally reported results, with the same taxonomic groups showing similar trends despite differences in data-processing methodologies. We usually found fewer significant ($q < 0.05$) differences between control and diseased groups, which is likely due to our choice of a non-parametric statistical test (Kruskall-Wallis) paired with a multi-test correction (FDR). Thus, our results are more conservative. We also collapsed to genus level in order to compare results across disparate studies, which prevented us from identifying species- or strain-specific associations which the original authors may have identified. A major advantage of our re-analysis is that each data set was processed and analyzed in the same way, which allowed us to more directly compare results across studies and diseases.

1.1 *Clostridium difficile* infection and enteric diarrhea are characterized by large-scale shifts in the microbiome (CDI; 4 studies)

Schubert et al. (2014) looked at how the gut microbiota differed between CDI patients with diarrhea ($n = 94$), non-CDI patients with diarrhea ($n = 89$), and non-diarrheal controls ($n = 155$).[1] Similar to other CDI studies, the authors found a significant reduction in alpha diversity in patients with diarrhea (Dunns multiple-comparison

test on AMOVA, $p < 0.0001$). They found that OTUs from the *Ruminococcaceae*, *Lachnospiraceae*, *Bacteroides*, *Prevotellaceae*, and *Porphyromonadaceae* families were enriched in healthy subjects relative to patients with CDI and non-CDI diarrhea. They also showed that OTUs from the *Enterococcus* genus and the *Enterobacteriaceae* and *Erysipelotrichaceae* families were more prevalent in patients with diarrhea. In our analysis of the data, we also observed a significant reduction in alpha diversity in patients with diarrhea ($q <= 0.05$, KW test). Similarly, we found that *Enterobacteriaceae*, *Enterococcus*, and *Erysipelotrichaceae* were enriched in CDI patients, in addition to *Fusobacterium*, *Parvimonas*, *Veillonella*, *Carnobacterium*, *Streptococcus*, *Tetragenococcus*, *Lactobacillus*, *Pediococcus*, *Gemella*, *Staphylococcus*, *Butyricicoccus*, *Robinsoniella*, *Clostridium XlVa*, *Clostridium XlVb*, *Ruminococcus2*, *Flavonifractor*, *Gemmiger*, *Mogibacterium*, *Peptostreptococcus*, *Clostridium XI*, *Eggerthella*, *Atopobium*, *Actinomyces*, *Arthrobacter*, *Aggregatibacter*, *Pseudomonas*, and *Dysgonomonas*. As in the original study, we found that *Bacteroides*, *Alistipes*, *Anaerovorax*, *Oxalobacter*, *Bordetella*, *Prevotellaceae*, *Porphyromonadaceae*, *Lachnospiraceae*, and *Ruminococcaceae* were more abundant in the healthy controls. We also found *Turicibacter*, *Dialister*, *Eubacterium*, *Asteroleplasma*, *Cloacibacillus*, *Bordetella*, *Oxalobacter*, *Sutterella*, *Parasutterella*, *Desulfovibrio*, *Sediminibacterium*, and *Methanobrevibacter* to be enriched in the controls ($q <= 0.05$, KW tests). Overall, our analysis closely matched what was presented in the original manuscript.

Vincent et al. (2013) compared 25 patients with CDI to 25 healthy control patients.[2] The authors found a significant reduction in alpha diversity ($p <= 0.05$, Mann-Whitney U test). They also report a reduction in *Bacteroidaceae* and *Clostridiales Incertae Sedis XI* in CDI patients relative to controls, and an enrichment in *Enterococcaceae* in CDI patients ($p < 0.05$, logistic regression). After reprocessing these data and collapsing abundances to the genus level, we observed a similar reduction in alpha diversity ($q <= 0.05$, KW test). We saw that the *Enterococcaceae* genera *Enterococcus* and *Proteus* were enriched in CDI patients. Healthy controls showed higher levels of *Fusobacterium*, *Peptoniphilus*, *Murdochiella*, *Anaerococcus*, *Finegoldia*, *Odoribacter*, *Prevotella*, and *Parabacteroides* relative to CDI patients. In summary, our results are fairly similar to the authors' original analysis, showing a depletion in *Bacteroidetes* and an enrichment in *Proteobacteria* in CDI patients.

Youngster et al. (2014) applied fecal microbiota transplants (FMTs) with materi-

als collected from 5 healthy donors to 20 patients with recurrent *Clostridium difficile* infections (CDIs).[3] The goal of this study was to determine whether nasal-gastric tube or colonoscopy administration of FMTs was most effective for treating CDIs (i.e. half of the CDI patients received one or the other treatment). The authors reported a significant reduction in alpha diversity in CDI patients vs. the healthy donors ($p < 0.001$, Mann-Whitney test). They did not assess whether there were significant differences in microbial community composition between CDI patients and donors, although they show that composition becomes more similar to donors following FMT. In our analysis, we also found a significant reduction in alpha diversity ($p <= 0.05$, KW test). *Enterococcus* was enriched in CDI patients relative to healthy stool donors ($q <= 0.05$, KW tests) and 15 genera were depleted in CDI patients relative to healthy controls. Healthy donors were enriched in genera from *Ruminococcaceae* and *Lachnospiraceae* families, in addition to the genera *Dialister* and *Anaerosporobacter*.

Singh et al. (2015) examined differences in the gut microbiome between individuals with enteric infections (n=200) and healthy controls (n=75).[4] The authors report a significant drop in alpha diversity in diseased patients relative to the controls (unknown test). They also report a general reduction in the dominance of *Firmicutes* and *Bacteroidetes* phyla and an increase in the prevalence of *Proteobacteria* in diseased patients. Specifically, they report an increase in the abundance of *Enterobacteriaceae*, *Lactobacillaceae*, *Pasteurellaceae*, *Streptococcus*, *Bacilli*, *Escherichia*, *Haemophilus*, and certain *Ruminococcus* species in patients with diarrhea. In healthy people, they report a significant enrichment in *Verrucomicrobia*, *Dorea*, *Blautia*, *Holdermania*, *Ruminococcaceae*, *Lachnospiraceae*, *Butyricimonas*, *Faecalibacterium*, *Bacteroidaceae*, and *Bifidobacterium*, *Sutterella*, *Parabacteroides*, *Rikenellaceae*, and *Oscillospira*. After re-processing the data, we found very similar results to those originally reported. We found that alpha diversity was significantly lower in patients with enteric infections ($q <= 0.05$, KW test). We saw significant enrichment in *Proteobacteria* families in patients with diarrhea, including *Enterobacteriaceae*, *Pasteurellaceae*, *Campylobacteraceae*, and *Neisseriaceae*. We also saw higher levels of *Fusobacterium*, *Parvimonas*, *Veillonella*, *Lactococcus*, *Streptococcus*, *Enterococcus*, *Tetragenococcus*, *Gemella*, *Ruminococcus II*, *Peptostreptococcus*, and *Collinsella* in diseased patients. In the healthy controls, we found enrichment of 43 genera, including *Sutterella*, *Verrucomicrobia* (*Akkermansia*), *Ruminococcaceae*, *Lachnospiraceae*, *Bacteroidaceae*, and

Bifidobacterium. In addition, we saw higher levels of several members of *Rumminococcaceae*, *Lachnospiraceae*, and *Bacteroidales* in healthy controls (q <= 0.05, KW tests). Overall, our results largely overlap with those presented, but we identify a number of significant taxa that were not originally reported.

Taken together, we see large-scale shifts in the microbiome associated with both CDI and non-CDI diarrhea. The dysbiosis of enteric infection and diarrhea is quite consistent across studies. In general, *Proteobacteria* increase in prevalence in patients with diarrhea, with a concomitant decrease in *Bacteroidetes* and *Firmicutes.* In particular, we see a reduction in butyrate-producing Clostridia, including genera within *Ruminococcaceae* and *Lachnospiraceae* families, which have been associated with a healthy gut. We also see in increase in prevalence of organisms often associated with lower pH and higher oxygen levels of the upper-gut, like *Lactobacillaceae* and *Enterobacteriaceae*,[5] in patients with diarrhea. Thus, diarrhea leads to consistent and large-scale rearrangements in the composition of the gut microbiome.

1.2 Colorectal cancer has a consistent, potentially pathogenic microbial signature (CRC; 4 studies)

Baxter et al. (2016) looked at differences in the microbiomes of 120 colorectal cancer (CRC) patients, 198 patients with non-cancerous adenomas, and 172 healthy controls.[6] Similar to prior work, the authors found that *Porphyromonas*, *Peptostreptococcus*, *Parvimonas*, and *Fusobacterium* were positively associated with CRC (random forest classifiers). Furthermore, they found that the absence of certain *Lachnospiraceae* species was associated with the presence of adenomas. We found similar patterns in our re-analysis of these data, with *Fusobacterium*, *Peptostreptococcus*, *Parvimonas*, and *Porphyromonas* enriched in CRC patients (q <= 0.05, KW tests). We also found higher levels of *Victivallis*, *Peptoniphilus*, *Anaerococcus*, *Catenibacterium*, *Staphylococcus*, *Collinsella*, *Enterobacter*, and *Alloprevotella* in CRC patients (q <= 0.05, KW tests). We found that healthy controls were enriched in *Lachnobacterium* (genus within *Lachnospiraceae*), *Gemmiger* (within *Rumminococcaceae*), *Clostridium XVIII*, and *Haemophilus* (q <= 0.05, KW tests). Overall, these results match what has been reported previously for CRC.[7]

Zeller et al. (2014) collected microbiome data from 41 CRC patients and 75

control patients.[8] At the phylum level, they found that *Proteobacteria*, *Fusobacteria*, and *Bacteroidetes*, were more abundant in CRC patients, while *Firmicutes* and *Actinobacteria* were enriched in control patients. At the genus level, the authors report higher levels of *Fusobacterium*, *Pseudoflavonifractor*, *Peptostreptococcus*, *Leptotrichia*, *Porphyromonas*, *Desulfovibrio*, *Parvimonas*, *Selenomonas*, and *Bilophila* in CRC patients (q <= 0.1, FDR-corrected Wilcoxon tests). Healthy controls were enriched in *Bifidobacterium*, *Acinetobacter*, *Campylobacter*, *Ruminococcus*, and *Eubacterium* genera (q <= 0.1, FDR-corrected Wilcoxon tests). In our re-analysis we found enrichment of *Fusobacterium*, *Parvimonas*, *Flavonifractor*, *Anaerotruncus*, *Anaerovorax*, *Peptostreptococcus*, *Comamonas*, *Eikenella*, *Butyricimonas*, and *Porphyromonas* genera in CRC patients (q <= 0.05, KW tests). In healthy patients, we found higher levels of *Anaerostipes* (within *Lachnospiraceae*; q <= 0.05, KW tests).

Wang et al. (2011) analyzed a cohort of 46 CRC patients and 56 healthy controls.[9] The authors found no difference in alpha diversity between CRC and control patients. CRC patients had higher abundances of *Porphyromonas*, *Escherichia-Shigella*, *Enterococcus*, *Streptococcus*, and *Peptostreptococcus* genera (p <= 0.05, Mann-Whitney). The authors report that healthy controls were enriched *Bacteroides*, *Roseburia*, *Alistipes*, *Eubacterium*, and *Parasutterella* genera (p <= 0.05, Mann-Whitney). We found very similar results in our re-analysis of these data. We saw greater levels of *Enterococcus*, *Peptostreptococcus*, *Enterobacter*, *Klebsiella*, *Escherichia-Shigella*, and *Porphyromonas* genera in CRC patients (q <= 0.05, KW tests). And we observed significantly higher levels of *Bacteroides*, and several genera within *Lachnospiraceae* in healthy controls (q <= 0.05, KW tests). Furthermore, we also did not detect any significant differences in alpha diversity between CRC and healthy patients.

Chen et al. (2012) analyzed stool from 22 healthy patients and 21 CRC patients.[10] The authors found that *Paraprevotella*, *Eubacterium*, *Desulfovibrio*, *Mogibacterium*, *Collinsella*, *Anaerotruncus*, *Slackia*, *Anaerococcus*, *Porphyromonas*, *Fusobacterium*, and *Peptostreptococcus* genera were significantly enriched in CRC patients relative to controls, while *Bifidobacterium*, *Faecalibacterium*, and *Blautia* were reduced in CRC patients (p <= 0.05, Mann-Whitney). In our re-analysis of this data set, we found no significant differences between CRC and control patients. Again, this is likely due to the small number of replicates and our implementation of multiple-test corrections. However, non-significant trends were largely in agreement with the original results.

Across these four colorectal cancer studies, we find significant agreement. Dysbiosis associated with CRC is generally characterized by increased prevalence of *Fusobacterium*, *Porphyromonas*, *Peptostreptococcus*, *Parvimonas*, *Leptotrichia*, *Desulfovibrio*, and *Anaerococcus* genera (i.e. these genera were higher in CRC patients in 2 or more studies). In addition, there is a consistent decrease in the abundances of *Faecalibacterium*, *Blautia*, *Bacteroides* genera and organisms from the *Lachnospiraceae* family in CRC patients. CRC appears to have a smaller impact on overall community structure than diahrrea. Indeed, we saw no significant differences in alpha diversity between healthy controls and CRC patients. In summary, CRC is characterized by a consistent enrichment of disease-associated bacteria.

1.3 Inflammatory bowel disease is characterized by a depletion of health-associated bacteria (IBD - ulcerative colitis and Crohn's disease; 4 studies)

Gevers et al. (2014) looked for microbial signatures of Crohn's disease (CD) samples across 447 CD patients and 221 non-IBD controls.[11] Non-IBD controls were patients with non-inflammatory conditions such as abdominal pain and diarrhea. The authors report increased abundance of *Enterobacteriaceae*, *Pasteurellaceae*, *Veillonellaceae*, and *Fusobacteriaceae* in CD patients. CD patients also showed a drop in the abundances of *Erysipelotrichales*, *Bacteroidales*, and *Clostridiales* (*Ruminococcaceae* and *Lachnospiraceae*) taxa. These results were based on a mixture of 16S amplicon and shotgun metagenomic sequencing. In our re-analysis of the 16S stool data, we found significant enrichment in *Anaerosporobacter*, *Roseburia*, *Hespellia*, *Ruminococcus II*, *Eubacterium*, *Pseudoflavonifractor*, *Sporobacter*, *Ruminococcus*, *Subdoligranulum*, *Papillibacter*, *Collinsella*, and *Methanobrevibacter* in healthy patients ($q <= 0.05$, KW tests). The only genera that we saw significantly enriched in CD patients were *Lactobacillus* and *Acetanaerobacterium* ($q <= 0.05$, KW tests). We found a similar set of taxa enriched in the controls, but did not detect as many significant CD-enriched genera as the authors reported. This is likely due to the fact that we restricted our analysis to the 16S stool data. However, we saw non-significant trends in *Enterobacteriaceae* and *Veillonellaceae* consistent with the results reported in the original paper.

Morgan et al. (2012) studied a cohort of 119 CD patients, 74 UC patients, and 27 healthy controls.[12] The authors found that healthy patients gut microbiomes were significantly enriched in *Roseburia*, *Phascolarctobacterium*, and an unclassified genus in the family *Veillonellaceae* (multivariate linear model, q <= 0.25). Patients with UC showed significantly higher levels of *Clostridiaceae* (multivariate linear model, q <= 0.25). In our re-analysis, we did not find any genera that were significantly enriched in IBD patients. We found that healthy patients had significantly greater abundances of *Ruminococcus*, and *Gemmiger* relative to both UC and CD patients (q <= 0.05, KW tests). Additionally, CD patients were depleted in *Clostridium IV* relative to healthy controls (q <= 0.05, KW tests).

Papa et al. (2012) studied a cohort of 23 CD patients, 43 UC patients, and 24 non-IBD controls.[13] Non-IBD controls were patients with symptoms such as: constipation, abdominal pain, gastroesophageal reflux, poor weight gain, diarrhea, blood in stool and oropharyngeal dysphagia. At the genus level, they found that controls were enriched in *Alistipes*, *Subdoligranulum*, *Anaerovorax*, *Oscillibacter*, *Parabacteroides*, *Odoribacter*, *Ruminococcus*, *Butyricicoccus*, *Akkermansia*, *Anaerotruncus*, *Sporobacter*, *Phascolarctobacterium*, *Lawsonia*, *Ethanoligenens*, *Peptococcus* relative to IBD patients (KW, $q < 0.01$). The only genus that was found to be enriched in IBD patients was *Escherichia-Shigella*. In our re-analysis, we also found *Escherichia-Shigella* and *Cronobacter* to be enriched in patients with IBD (q <= 0.05, KW tests). When comparing healthy controls with UC patients, we also found an enrichment of *Haemophilus* in the UC patients. Control patients showed higher abundances of *Phascolarctobacterium*, *Butyricicoccus*, *Ruminococcus II*, *Oscillibacter*, *Ruminococcus*, *Gemmiger*, *Subdoligranulum*, *Clostridium IV*, *Odoribacter*, *Alistipes*, and *Parabacteroides* relative to all IBD patients (q <= 0.05, KW tests). Additionally, control patients were enriched in *Clostridium XIVa*, *Flavonifractor*, and *Akkermansia* relative to UC patients. Overall, our results match very closely what was found in the original paper.

Willing et al. (2010) compared 29 CD patients and 16 UC patients to 35 healthy controls.[14] The authors reported variable, and sometimes opposing shifts in the microbiomes of patients with UC, ileal CD and colonic CD at different taxonomic resolutions. We found no significant differences between IBD and healthy patients in our re-analysis. When comparing healthy controls with CD cases only, we found an

enrichment of *Butyricicoccus* and *Oscillibacter* in the control patients (q <= 0.05, KW tests).

In summary, there are certain consistencies across IBD studies. IBD patients tend to be depleted in butyrate-producing clostridia: *Ruminococcus* and *Lachnospiraceae.* The organisms the are enriched in CD and UC patients tend to vary across studies. One consistency is organisms associated with the upper gut, like *Lactobacillus* and *Enterobacteriaceae* appear to be enriched in IBD patients.[5] This result fits with the reduced stool transit times associated with IBD (i.e. diarrhea).

1.4 Obesity shows a somewhat inconsistent microbial signature (OB; 5 studies)

Goodrich et al. (2014) studied a cohort of 416 twin pairs: 422 normal BMI, 322 overweight, and 185 obese.[15] The authors report higher levels of *Lactobacillaceae*, *Eggerthella*, and *Lachnospiraceae* (*Blautia* and *Dorea*) in obese individuals (q < 0.05, FDR-corrected T-test). They showed enrichment for *Christensenellaceae*, *Dehalobacterium*, *Lachnospira*, *Mogibacteriaceae*, *Rikenellaceae*, *Methanobre*, *Coriobacteriaceae*, *Peptococcaceae*, *Oscillospira*, *Ruminococcaceae*, and *Sarcina* in healthy BMI individuals (q < 0.05, FDR-corrected T-test). In our re-analysis, we found higher levels of *Streptococcus*, *Weissella*, *Roseburia*, *Blautia*, *Clostridium XlVb*, and *Mogibacterium* in obese individuals, while *Robinsoniella*, *Ruminococcaceae* (*Oscillibacter*, *Pseudoflavonifractor*, *Sporobacter*, and *Anaerofilum*), and *Anaerovorax* were more abundant in low-BMI individuals (q <= 0.05, KW tests). Our results only partially agree with the authors' original findings, which may be due to the fact that we used a different statistical test and OTU-calling method and that we binned the data at the genus level.

Zupancic et al. (2012) analyzed 310 individuals from an Amish population with varying BMIs.[16] They found a significant positive correlation between the abundance of *Collinsella* and BMI (i.e. enriched in obese individuals), while *Lachnobacterium*, *Anaerotruncus*, *Faecalibacterium*, and *Clostridium* were negatively correlated with BMI (i.e. enriched lean individuals) (p ¡ 0.001, Spearman correlation). We found no significant differences in the proportion of genera between lean and obese individuals in our re-analysis.

Turnbaugh et al. (2008) looked differences in gut microbial community structure between 31 monozygotic and 23 dizygotic twin pairs concordant for leanness or obesity.[17] The authors report a reduction in alpha diversity in obese individuals. They also report a significant decrease in *Bacteroidetes* and an increase in *Actinobacteria* in obese twins. In our re-analysis of these data, we did not see a significant reduction in alpha diversity (Supplementary Figure 6). We found significant increases in *Catenibacterium*, *Acidaminococcus*, *Megasphaera*, *Lactobacillus*, *Roseburia*, and *Collinsella* in obese twins (q <= 0.05, KW tests). *Coprobacillus*, *Clostridium XVIII*, *Phascolarctobacterium*, *Clostridium XlVb*, *Oscillibacter*, *Flavonifractor*, *Pseudoflavonifractor*, *Ruminococcus*, *Clostridium IV*, *Gordonibacter*, *Alistipes*, and *Barnesiella* were significantly enriched in lean twins (q <= 0.05, KW tests).

Ross et al. (2015) looked at 63 Mexican American patients with varying BMIs.[18] They found no significant differences between patients with high and low BMIs within their 63 patient cohort, but identified several significant differences between their patient population and the HMP data set. However, it is unclear whether these differences were related to obesity, so we do not discuss them here. Our re-analysis of these results also found no significant differences in the relative abundances of bacterial genera between high- and low-BMI subjects.

Zhu et al. (2013) compared across a cohort of 16 healthy and 25 obese patients, in addition to 22 patients with Nonalcoholic steatohepatitis (see below).[19] For obesity, the authors found that *Prevotella* was enriched in high-BMI patients, while healthy controls showed significantly greater relative abundances of *Bifidobacterium*, *Blautia*, and *Faecalibacterium* (p <= 0.05, ANOVA with post-hoc Tukey's tests). In our re-analysis of these data, we found a significant enrichment of *Peptoniphilus*, *Anaerococcus*, *Finegoldia*, *Leuconostoc*, *Mogibacterium*, *Varibaculum*, *Campylobacter*, *Prevotella*, and *Porphyromonas* in obese patients (q <= 0.05). Healthy patients were significantly enriched in *Akkermansia*, *Murdochiella*, *Blautia*, *Lachnospiracea incertae sedis*, and *Clostridium IV*, *Anaerovorax* (q <= 0.05, KW tests).

Overall, we found several differences between lean and obese patients that were consistent across at least two studies. *Roseburia* and *Mogibacterium* were enriched in obese individuals in more than one study. *Pseudoflavonifractor*, *Oscillobacter*, *Anaerovorax* and *Clostridium IV* were enriched in the controls across more than one study. However, no genera showed consistent differences across three or more studies.

Our results are largely consistent with a recent meta-analysis of obesity studies, which found no universal signature of human obesity.[20]

1.5 Human immunodeficiency virus microbial signature is confounded with patient cohorts (HIV; 3 studies)

Dinh et al. (2015) compared the gut microbiome from 16 healthy patients to 22 patients with chronic HIV infections.[21] The authors report an general enrichment in *Proteobacteria* in HIV-infected patients. At the genus level, they found a significant enrichment in *Barnesiella* and a depletion in *Alistipes* in HIV-infected patients (LEfSe, $p < 0.05$). In our re-analysis of these data we found no significant differences in the relative abundances of genera between healthy and HIV-infected patients.

Lozupone et al. (2013) looked at 22 HIV-positive patients and 13 healthy controls.[22] The authors reported enrichment of *Prevotella*, *Catenibacterium*, *Dialister*, *Allisonella*, and *Megasphera* genera in HIV-positive patients, while *Bacteroides* and *Alistipes* were more abundant in controls ($p < 0.05$, ANOVA). We found all the associations reported above in our re-analysis. Additionally, we saw higher relative abundances of *Erysipelotrichaceae incertae sedis*, *Peptococcus*, *Mogibacterium*, *Peptostreptococcus*, *Desulfovibrio*, *Hallella*, and *Alloprevotella* in HIV-positive patients. And healthy patients were also enriched in *Oridibacter*, *Anaerostipes*, and *Parasutterella*. Many of the significant genera from the Lozupone study were shown to be strongly associated with sexual behavior in the Noguera-Julian study (i.e. these genera were significantly different in men who have sex with men versus other subjects; see below) and may not necessarily be related to HIV status.

Noguera-Julian et al. (2016) studied a cohort of 293 HIV-infected patients and 57 healthy controls. The authors found that many putative associations between HIV and the microbiome were driven by sexual preference (i.e. *Prevotella*, along with several other genera, were enriched in men who have sex with men (MSM)). After controlling for this demographic confounder, the authors reported that they were not able to classify HIV positive and negative patients MSM patients. Due to the large size of their study, the authors were able to separate the influences of sexual behavior and HIV-status from one another and found that the majority of reported HIV-associations are likely confounded with sexual behavior.

Overall, there is not yet a strong consensus on the impacts of HIV on the human gut microbiome. Differences between patient cohorts may have obscured any putative HIV signal across studies. For example, all the patients in the Dinh et al. (2015) study were on antiretroviral therapy (ART), while only some of the patients in the other two studies were on ART. Noguera-Julian et al. (2016) found that patients who initiated ART within the first 6 months of HIV infection were able to maintain gut microbial community richness, unlike patients that were not on ART. In addition, the Noguera-Julian et al. (2016) paper was able to show that prior results showing enrichment of *Prevotella* in HIV-positive patients was an artifact due to this genera being enriched in men who have sex with men.

1.6 Autism spectrum disorder (ASD; 2 studies)

Kang et al. (2013) reported a reduced prevalence of *Prevotella* and other fermentative organisms in the guts of ASD children.[23] In particular, the authors showed significant ($q <= 0.05$, Mann-Whitney) depletion in unclassified *Prevotella* and *Veillonellaceae* genera in autistic children ($n = 20$ treatment and 20 controls). The authors also note a reduced alpha diversity in autistic children. After reprocessing these data, we found no significant differences in alpha diversity or genera abundances between autistic and control children (Figure 1; $q > 0.05$, Kruskal-Wallis). The original conclusion that *Prevotella* and *Veillonellaceae* were different was based on q-values of 0.04, which is only moderately convincing evidence against the null-hypothesis. Therefore, the loss of this marginal significance (for $q <= 0.05$) is unsurprising when using a different statistical test.

In a more recent study, Son et al. (2015) found no significant differences in microbial community diversity or composition between autistic and neurotypical children ($n = 59$ ASD and 44 neurotypical).[24] One genus, representing chloroplast sequences, was associated with ASD children with functional constipation, but this signal appeared to be due to dietary intake of chia seeds. Similar to the authors findings, we did not detect any significant differences in genera abundances between ASD children and neurotypical children in the reprocessed data ($q > 0.05$, Kruskal-Wallis).

Taken together, we find no evidence for changes in the composition or diversity of the gut microbiome in response to ASD. However, we cannot discount subtle dysbiosis (i.e. small effect size) in response to ASD due to the small number of patients in each

study.

1.7 Type 1 Diabetes (T1D; 2 studies)

Alkanani et al. (2015) compared 23 healthy patients to 35 early-onset T1D patients and 21 seropositive T1D patients.[25] The authors report higher relative abundances of *Lactobacillus*, *Prevotella* and *Staphylococcus* genera in healthy patients ($p < 0.05$, Wilcoxon). T1D patients showed higher levels of *Bacteroides* ($p < 0.05$, Wilcoxon). In our re-analysis, we found no significant differences in bacterial genera across healthy and diseased patients.

Mejia-Leon et al. (2014) compared 8 healthy patients to 8 early-onset T1D patients and 13 T1D patients who had received 2 years of treatment.[26] Similar to Alkanani et al. (2015), they found controls to be significantly enriched in *Prevotella* and T1D patients enriched in *Bacteroides* ($p < 0.05$, ANOVA, Tukey-Kramer test). They also found higher levels of *Acidaminococcus* and *Megamonas* genera (in the *Veillonellaceae* family) in the controls ($p < 0.05$, ANOVA, Tukey-Kramer test). We saw no significant differences in our re-analysis of these data.

Overall, the original authors report a consistent increase in *Bacteroides* and depletion in *Prevotella* genera associated with T1D. However, our re-analysis found that these differences did not pass our significance threshold. Thus, we cannot yet conclude that there is a consistent dysbiosis associated with T1D.

1.8 Nonalcoholic steatohepatitis (NASH; 2 studies)

Zhu et al. (2013) compared the microbiomes from 16 healthy individuals to 22 patients with NASH.[19] They found significantly lower relative abundances of *Bifidobacterium*, *Blautia*, and *Faecalibacterium* genera in NASH patients ($p <= 0.05$, ANOVA with post-hoc Tukey's tests). NASH patients were enriched in *Escherichia*, compared to controls, and tended to show increased levels of *Proteobacteria* ($p <= 0.05$, ANOVA with post-hoc Tukey's tests). In our re-analysis, we found that NASH patients showed significantly higher levels of *Fusobacterium*, *Peptoniphilus*, *Anaerococcus*, *Finegoldia*, *Gallicola*, *Negativicoccus*, *Leuconostoc*, *Weissella*, *Lactobacillus*, *Peptococcus*, *Moryella*, *Syntrophococcus*, *Mogibacterium*, *Olsenella*, *Varibaculum*, *Mobiluncus*, *Pyramidobacter*, *Escherichia/Shigella*, *Campylobacter*, *Hallella*, *Prevotella*,

and *Porphyromonas* genera ($q < 0.05$, KW test). Conversely, control patients were significantly enriched in *Akkermansia*, *Murdochiella*, *Coprococcus*, *Anaerostipes*, *Blautia*, *Lachnospiracea incertae sedis*, *Faecalibacterium*, *Ruminococcus*, *Gemmiger*, *Clostridium IV*, *Anaerovorax*, *Clostridium XI*, *Corynebacterium*, *Bifidobacterium*, *Alistipes*, and *Barnesiella* genera ($q < 0.05$, KW test).

Wong et al. (2013) investigated a cohort of 16 healthy and 22 NASH patients.[27] They found that control patients were enriched in *Faecalibacterium* and *Anaerosporobacter* genera, while NASH patients showed significantly higher levels of *Parabacteroides* and *Alisonella* genera ($p < 0.05$, t-test). In our re-analysis of these data, we saw no significant differences.

In summary, there were not many consistencies between the two NASH studies analyzed here. The original studies consistently report a depletion in *Faecalibacterium* in NASH patients. Thus, the overall influence of NASH on the microbiome is difficult to assess without further study.

1.9 Minimal hepatic encephalopathy and liver cirrhosis (LIV; 1 study)

Zhang et al. (2013) looked at the microbiomes of 26 healthy patients, 26 patients with MHE, and 25 patients with CIRR.[28] The original paper reported several genera that differed between diseased and control patients. *Odoribacter*, *Flavonifractor*, and *Coprobacillus* were all enriched in MHE patients relative to controls, while *Eubacterium*, *Lachnospira*, *Parasutteralla*, and an unclassified *Erysipelotrichaceae* genus were enriched in healthy patients ($p < 0.01$, Mann-Whitney). The authors also reported depletion in *Prevotella* in non-MHE patients with cirrhosis (CIRR), relative to controls. When we re-processed and re-analyzed these data, the only difference we found was an enrichment in *Veillonella* in case (MHE and CIRR) patients ($q < 0.05$, KW test). When comparing controls with MHE patients alone, we also saw an enrichment of *Faecalibacterium* in healthy controls relative to MHE cases.

1.10 Rheumatoid and psoriatic arthritis (ART; 1 study)

Scher et al. (2013) investigated the impacts of arthritis on a cohort of 86 arthritic and 28 healthy patients.[29] The authors report that greater abundances of *Prevotella*

copri can predict susceptibility to arthritis. There were three types of arthritic conditions studied, but only new-onset untreated rheumatoid arthritis (NORA) showed a strong association with multiple *Prevotella* OTUs among others ($q < 0.01$, LEfSe). The other RA groups were not easily distinguishable from controls. Indeed, when grouping all arthritis patients together for our re-analysis as well as comparing RA and psoriatic arthritis patients separately, we did not find any genera that were significantly different between arthritic patients and controls.

1.11 Parkinson's disease (PAR; 1 study)

Scheperjans et al. (2014) looked for differences in the gut microbiome between 72 neurotypical patients and 72 Parkinson's (PAR) patients.[30] They found a small handful of significant differences at the family level. Control patients showed higher relative abundances of *Prevotellaceae*, while PAR patients were enriched in *Lactobacillaceae*, *Verrucomicrobiaceae*, *Bradyrhizobiaceae*, and *Clostridiales Incertae Sedis* ($q < 0.05$, Mann-Whitney). In our re-analysis, we found significantly higher relative abundances of *Lactobacillus* (within *Lactobacillaceae*) and *Alistipes* (within *Rikenellaceae*) in PAR patients ($q < 0.05$, KW tests).

Supplementary Note 2: Stratifying heterogenous case groups shows consistent disease-specific signals

In our main analyses, we combined Crohn's disease (CD) and ulcerative colitis (UC) patients together as IBD cases. We also performed separate analyses on these individual patient groups. All four IBD studies included CD cases and three included UC cases (all except Gevers et al. (2014)[11]). We performed the same analysis as in Figure 1 for these stratified groups, and found that both CD and UC patients are characterized by depletion of similar health-associated microbes (Supplementary Figures 4 and 5). Interestingly, neither UC nor CD seemed to have a larger microbiome shift: only one dataset for each type of comparison had more than 10 significant genera (Gevers et al. (2014), 14 CD-associated genera; Papa et al. (2012), 17 UC-associated genera). Additional studies comparing UC- and CD-specific microbiome alterations will be needed to tease out whether and how these IBD subtypes differentially impact

the gut microbiome.

We also performed stratified analyses on the arthritis (ART) and liver (LIV) patients in the Scher et al. (2013) and Zhang et al. (2013) datasets, respectively[28,29] (Supplementary Figure 4). The random forest classifiers performed similarly well on the stratified patient groups than on the combined cases. As in the combined analyses, neither type of arthritis (rheumatoid arthritis (RA) or psoriatic arthritis (PSA)) had any significant genus-level associations. In the Zhang et al. (2013) dataset, 1 genus was significantly associated with the liver cirrhosis (CIRR) patients and 2 with the minimal hepatic encelopathy (MHE) patients. As in the original combined analysis, *Veillonella* was associated with both groups of patients. In our stratified analysis, *Faecalibacterium* was additionally significantly associated with non-MHE healthy controls. However, the lack of other arthritis or liver datasets in this analysis prevents us from drawing more generalized conclusions from these stratified analyses.

Supplementary Note 3: Healthy vs. disease classifier identifies general microbiome shifts

To further address the question of whether we could find a robust, generalized signal for diseased microbiomes regardless of the disease type, we built two classifiers to distinguish healthy patients from any type of case patients. In these classifiers, we excluded the two datasets which did not have healthy controls (Gevers et al. (2014)[11] and Papa et al. (2012),[13] which used non-IBD patients as controls) and CDI Youngster (2014),[3] which had only 4 distinct controls. First, we performed leave-one-dataset-out cross-validation to determine whether a general healthy vs. disease classifier trained on the other datasets could still classify cases from controls in a test dataset. These AUCs correlated well with the single-dataset classifiers, though usually performed slightly less well than the single-dataset classifiers (Pearson $\rho = 0.56$, p = 0.003; Supplementary Figure 9). We also built a more stringent leave-one-disease-out classifier to ensure that the diarrhea datasets and others with strong microbiome signals were not driving the classification ability of all other diseases. Surprisingly, this classifier performed similarly to the leave-one-dataset-out classifier (Supplementary Figure 9). The positive correlation with the original single-dataset classification results (Pearson $\rho = 0.47$, p = 0.02) indicates that there is a generalizable healthy vs.

disease microbiome signal that is being identified even across different diseases. These results also indicate that models for each disease group are predictive of cases and controls for other datasets within that group, since the leave-one-dataset-out classifier, which included datasets of the test disease group in the training set, performed better than the leave-one-disease-out classifier, which did not.

Supplementary Note 4: Shared microbial response is robust to different definitions

Our simple heuristic defined non-specific microbes as those which were significantly enriched or depleted in two diseases. To ensure that this definition was not being dominated by the diarrhea datasets and that we were indeed identifying microbes which respond non-specifically to multiple diseases, we re-defined the non-specific genera as those which were significantly enriched or depleted in two diseases, excluding datasets with diarrhea cases (Schubert et al. (2014),[1] Singh et al. (2015),[4] Vincent et al. (2013),[2] and Youngster et al. (2014)[3]). We found that 27 out of the 51 original non-specific genera were recovered, with all health- and disease-associated effects in matching directions (Supplementary Figure 10). Thus, the majority of the shared microbial response is robust to the exclusion of diarrhea datasets.

We also re-defined non-specific microbes using Stouffer's method to combine p-values across all datasets (except Papa et al. (2012),[13] Gevers et al. (2014),[11] and Lozupone et. al (2013)[22]).[31] We combined each dataset's FDR-corrected q-values with `scipy.stats.combine_pvalues(method='stouffer')`, using the square root of each study's sample size as the weights. Genera with a combined q-value less than 0.05 were considered non-specific responders. Overall, these results did not conflict with the heuristic definition (i.e. only two genera, *Porphyromonas* and *Gemmiger*, were "health-associated" with one method and "disease-associated" with the other; Supplementary Figure 10). Stouffer's method is less conservative than the heuristic definition, identifying 111 genera in the non-specific response (60 health-associated and 51 disease-associated). In addition, using Stouffer's method does not allow for the identification of mixed genera (i.e. those which respond in both health- and disease-associated directions across multiple diseases). Finally, combining q-values with Stouffer's method does not ensure that identified microbes are responding non-

specifically to multiple diseases: one highly significant genus in a large study can dominate other q-values and be flagged as a non-specific responder, despite only being associated with one disease. Thus, the heuristic definition is more conservative and more directly related to the biological question of identifying shared microbial responses to disease.

We tested whether the overall number of non-specific responders we observed was greater than we would expect to see due to chance. We built an empirical null distribution of the number of each type of non-specific responder. We shuffled q-values within each dataset, re-defined non-specific responders, and counted how many health-associated, disease-associated, and mixed genera were found, repeating this process 1000 times. When we considered significance in two diseases as the threshold for our heuristic (as presented in the main text), we did not find a significantly larger number of non-specific responses than would be expected by chance (Supplementary Figure 11). When we raised the heuristic threshold to three diseases our results became more significant, but there was a large reduction in the number of identified non-specific genera. Thus, there is currently not enough information to fully distinguish between microbes that are sporadically detected across multiple diseases from those that may be consistently associated with general health or disease. Future meta-analyses that include many more datasets for each of many conditions might be able to distinguish microbes that are consistently associated with health or disease from those that are sporadically associated with different conditions.

Despite the fact that the number of non-specific microbes did not reach statistical significance, we identified multiple lines of evidence for a coherent microbial response to health and disease. First, the healthy vs. disease classifiers successfully classified case patients across a variety of diseases even when the disease being tested was not in the training set, indicating that some aspects of disease-associated microbiome shifts can generalize across diseases (Supplementary Figure 9). Second, the statistical significance of the number of non-specific responders increased as we increased the number of diseases threshold (Supplementary Figure 11). Thus, future meta-analyses which include many more studies and disease states may be able to more robustly identify bacteria which respond across a broader variety of disease states. Third, we saw a coherent phylogenetic signal in the non-specific response (e.g. Proteobacteria and *Lactobacillaceae* associated with disease and *Rumminococcaceae* and

Lachnospiraceae associated with health), which points to potential mechanisms (e.g. shorter stool transit time or inflammation) for a shared response to health or disease (Figure 3A). Thus, we expect that future meta-analyses that include more studies and diseases will identify a consistent set of bacteria that form a general microbial response to health and disease in the gut.

Dataset ID	Year	Controls	N (controls)	Cases	N (cases)	Median reads per sample	Sequencer	16S Region	Ref.
Scher 2013, ART	2013	H	28	PSA, RA	86	2194.0	454	V1-V2	29
Kang 2013, ASD	2013	H	20	ASD	19	1345.0	454	V2-V3	23
Son 2015, ASD	2015	H	44	ASD	59	4777.0	Miseq	V1-V2	24
Schubert 2014, CDI	2014	H	154	CDI	93	4897.0	454	V3-V5	1
Schubert 2014, nonCDI	2014	H	154	nonCDI	89	4903.0	454	V3-V5	1
Singh 2015, EDD	2015	H	82	EDD	201	2585.0	454	V3-V5	4
Vincent 2013, CDI	2013	H	25	CDI	25	2526.5	454	V3-V5	2
Youngster 2014, CDI	2014	H	4	CDI	19	15081.0	Miseq	V4	3
Baxter 2016, CRC	2016	H	172	CRC	120	9913.5	Miseq	V4	6
Chen 2012, CRC	2012	H	22	CRC	21	1152.0	454	V1-V3	10
Wang 2012, CRC	2012	H	54	CRC	44	161.0	454	V3	9
Zeller 2014, CRC	2014	H	75	CRC	41	120989.0	MiSeq	V4	8
Dinh 2015, HIV	2015	H	15	HIV	21	3248.5	454	V3-V5	21
Lozupone 2013, HIV	2013	H	13	HIV	23	3262.0	MiSeq	V4	22
Noguera-Julian 2016, HIV	2016	H	34	HIV	205	16506.0	MiSeq	V3-V4	32
Gevers 2014, IBD	2014	nonIBD	16	CD	146	9773.5	Miseq	V4	11
Morgan 2012, IBD	2012	H	18	UC, CD	108	1022.5	454	V3-V5	12
Papa 2012, IBD	2012	nonIBD	24	UC, CD	66	1323.5	454	V3-V5	13
Willing 2010, IBD	2009	H	35	UC, CD	45	1118.5	454	V5-V6	14
Zhang 2013, LIV	2013	H	25	CIRR, MHE	46	487.0	454	V1-V2	28
Wong 2013, NASH	2013	H	22	NASH	16	1980.0	454	V1-V2	27
Zhu 2013, NASH	2013	H	16	NASH	22	10863.0	454	V4	19
Goodrich 2014, OB	2014	H	428	OB	185	27077.0	Miseq	V4	15
Ross 2015, OB	2015	H	26	OB	37	4562.0	454	V1-V3	18
Turnbaugh 2009, OB	2009	H	61	OB	195	1556.5	454	V2	17
Zhu 2013, OB	2013	H	16	OB	25	9778.0	454	V4	19
Zupancic 2012, OB	2012	H	96	OB	101	1645.0	454	V1-V3	16
Scheperjans 2015, PAR	2015	H	74	PAR	74	2351.5	454	V1-V3	30
Alkanani 2015, T1D	2015	H	55	T1D	57	9117.0	MiSeq	V4	25
Mejia-Leon 2014, T1D	2014	H	8	T1D	21	4702.0	454	V4	26

Supplementary Table 1: Datasets collected and processed through standardized pipeline. Disease labels: ART = arthritis, ASD = austism spectrum disorder, CD = Crohn's disease, CDI = *Clostridium difficile* infection, CIRR = liver cirrhosis, CRC = colorectal cancer, EDD = enteric diarrheal disease, H = healthy, HIV = human immunodeficiency virus, LIV = liver diseases, MHE = minimal hepatic encephalopathy, NASH = non-alcoholic steatohepatitis, OB = obesity, PAR = Parkinson's disease, PSA = psoriatic arthritis, RA = rheumatoid arthritis, T1D = type I diabetes, UC = ulcerative colitis. nonCDI controls are patients with diarrhea who tested negative for *C. difficile* infection. nonIBD controls are patients with gastrointestinal symptoms but no intestinal inflammation. Datasets are ordered alphabetically by disease and within disease by first author.

Dataset ID	Data type	Barcodes	Primers	Quality filtering	Quality cutoff	Length trim
Scher 2013, ART	fastq	No	Yes	-fastq_truncqual	25	200
Kang 2013, ASD	fastq	No	Yes	-fastq_truncqual	25	200
Son 2015, ASD	fastq	No	Yes	-fastq_truncqual	25	200
Schubert 2014, CDI	fastq	No	Yes	-fastq_truncqual	25	150
Vincent 2013, CDI	fastq	No	Yes	-fastq_truncqual	20	101
Youngster 2014, CDI	fastq	No	No	-fastq_truncqual	25	200
Baxter 2016, CRC	fastq	No	No	-fastq_truncqual	25	250
Chen 2012, CRC	fastq	Yes	Yes	-fastq_truncqual	25	200
Wang 2012, CRC	fastq	Yes	Yes	-fastq_truncqual	25	150
Zeller 2014, CRC	fastq	No	No	-fastq_truncqual	25	200
Singh 2015, EDD	fasta	n/a	n/a	n/a	n/a	200
Dinh 2015, HIV	fastq	No	No	-fastq_truncqual	25	200
Lozupone 2013, HIV	fastq	No	No	-fastq_truncqual	25	150
Noguera-Julian 2016, HIV	fastq	No	Yes	-fastq_truncqual	25	200
Gevers 2014, IBD	fastq	No	No	-fastq_truncqual	25	200
Morgan 2012, IBD	fastq	No	Yes	-fastq_truncqual	25	200
Papa 2012, IBD	fasta	n/a	n/a	n/a	n/a	200
Willing 2010, IBD	fastq	No	Yes	-fastq_maxee	2	200
Zhang 2013, LIV	fastq	No	Yes	-fastq_truncqual	25	200
Wong 2013, NASH	fastq	No	No	-fastq_truncqual	25	200
Zhu 2013, NASH	fasta	n/a	n/a	n/a	n/a	200
Schubert 2014, nonCDI	fastq	No	Yes	-fastq_truncqual	25	150
Goodrich 2014, OB	fastq	No	No	-fastq_truncqual	25	200
Ross 2015, OB	fastq	No	No	-fastq_truncqual	25	150
Turnbaugh 2009, OB	fasta	n/a	n/a	n/a	n/a	200
Zhu 2013, OB	fasta	n/a	n/a	n/a	n/a	200
Zupancic 2012, OB	fastq	No	No	-fastq_truncqual	25	200
Scheperjans 2015, PAR	fastq	No	Yes	-fastq_truncqual	25	200
Alkanani 2015, T1D	fastq	No	No	-fastq_maxee	2	200
Mejia-Leon 2014, T1D	fastq	Yes	Yes	-fastq_truncqual	25	150

Supplementary Table 2: Processing parameters for all datasets. `Barcodes` column indicates whether we assigned reads to samples by their barcodes (`Yes`) or if the files were already de-multiplexed (`No`). `Primers` column indicates whether we removed the primers from sequences. `Quality filtering` and `Quality cutoff` columns indicate the type of quality filtering we performed on the data. `Length trim` is the length to which all sequences were truncated before clustering into OTUs. In the case of `-fastq_truncqual` quality filtering, reads were length trimmed after quality truncation. In the case of `-fastq_maxee` quality filtering, reads were length trimmed before quality filtering. Datasets are ordered alphabetically by disease and within disease by first author. ART = arthritis, ASD = autism spectrum disorder, CDI = *Clostridium difficile* infection, CRC = colorectal cancer, EDD = enteric diarrheal disease, HIV = human immunodeficient virus, IBD = inflammatory bowel disease, LIV = liver disease, NASH = non-alcoholic steatohepatitis, nonCDI = non-*Clostridium difficile* infection, OB = obesity, PAR = Parkinson's disease, T1D = type I diabetes.

Dataset ID	Raw data	Metadata
Scher 2013, ART	SRA study SRP023463	SRA
Kang 2013, ASD	SRA study SRP017161	SRA
Son 2015, ASD	SRA study SRP057700	SRA
Schubert 2014, CDI	mothur.org/CDI_MicrobiomeModeling	mothur.org/CDI_MicrobiomeModeling
Vincent 2013, CDI	email authors	email authors
Youngster 2014, CDI	SRA study SRP040146	email authors
Baxter 2016, CRC	SRA study SRP062005	SRA
Chen 2012, CRC	SRA study SRP009633	SRA sample description
Wang 2012, CRC	SRA study SRP005150	SRA study description
Zeller 2014, CRC	ENA study PRJEB6070	Table S1 and S2
Singh 2015, EDD	http://dx.doi.org/10.6084/m9.figshare.1447256	Additional File 4
Dinh 2015, HIV	SRA study SRP039076	SRA
Lozupone 2013, HIV	ENA study PRJEB4335	Qiita study 1700
Noguera-Julian 2016, HIV	SRA study SRP068240	SRA
Gevers 2014, IBD	SRA study SRP040765	Table S2
Morgan 2012, IBD	SRA study SRP015953	http://huttenhower.sph.harvard.edu/ibd2012
Papa 2012, IBD	email authors	email authors
Willing 2010, IBD	email authors	email authors
Zhang 2013, LIV	SRA study SRP015698	SRA
Wong 2013, NASH	SRA study SRP011160	SRA
Zhu 2013, NASH	MG-RAST, study mgp1195	MG-RAST
Schubert 2014, nonCDI	mothur.org/CDI_MicrobiomeModeling	mothur.org/CDI_MicrobiomeModeling
Goodrich 2014, OB	ENA studies PRJEB6702 and PRJEB6705	ENA
Ross 2015, OB	SRA study SRP053023	SRA
Turnbaugh 2009, OB	https://gordonlab.wustl.edu/NatureTwins_2008/TurnbaughNature_11_30_08.html	Table S1
Zhu 2013, OB	MG-RAST, study mgp1195 (same data as nash_zhu)	MG-RAST
Zupancic 2012, OB	SRA study SRP002465	SRA
Scheperjans 2015, PAR	ENA study PRJEB4927	sample names
Alkanani 2015, T1D	email authors	email authors
Mejia-Leon 2014, T1D	email authors	email authors

Supplementary Table 3: Locations of raw data and associated metadata for each dataset used in these analyses. Datasets are ordered alphabetically by disease and within disease by first author. ART = arthritis, ASD = autism spectrum disorder, CDI = *Clostridium difficile* infection, CRC = colorectal cancer, EDD = enteric diarrheal disease, HIV = human immunodeficient virus, IBD = inflammatory bowel disease, LIV = liver disease, NASH = non-alcoholic steatohepatitis, nonCDI = non-*Clostridium difficile* infection, OB = obesity, PAR = Parkinson's disease, T1D = type I diabetes.

Dataset ID	AUC	Fisher's p	Kappa score
Singh 2015, EDD	0.96	7.9e-31	0.7
Schubert 2014, CDI	0.99	8.7e-49	0.88
Schubert 2014, nonCDI	0.98	6.3e-38	0.79
Vincent 2013, CDI	0.91	1.6e-06	0.68
Goodrich 2014, OB	0.67	0.00014	0.11
Turnbaugh 2009, OB	0.84	1.7e-06	0.28
Zupancic 2012, OB	0.44	0.16	-0.11
Ross 2015, OB	0.49	0.75	-0.068
Zhu 2013, OB	0.86	1.3e-05	0.69
Baxter 2016, CRC	0.77	5.4e-16	0.43
Zeller 2014, CRC	0.82	3.4e-06	0.41
Wang 2012, CRC	0.9	2.6e-11	0.67
Chen 2012, CRC	0.78	0.034	0.35
Gevers 2014, IBD	0.71	1	0
Morgan 2012, IBD	0.81	0.0025	0.26
Papa 2012, IBD	0.84	0.0019	0.34
Willing 2010, IBD	0.66	0.81	0.026
Noguera-Julian 2016, HIV	0.67	1	0
Lozupone 2013, HIV	0.92	8.7e-06	0.76
Dinh 2015, HIV	0.22	0.062	-0.26
Son 2015, ASD	0.39	0.12	-0.16
Kang 2013, ASD	0.76	0.056	0.33
Alkanani 2015, T1D	0.71	0.0078	0.27
Mejia-Leon 2014, T1D	0.77	0.18	0.25
Wong 2013, NASH	0.68	0.098	0.28
Zhu 2013, NASH	0.93	1.3e-07	0.84
Scher 2013, ART	0.62	1	-0.034
Zhang 2013, LIV	0.8	0.016	0.29
Scheperjans 2015, PAR	0.67	0.0083	0.23

Supplementary Table 4: Area under the ROC curve (AUC), Fisher's p-values, and Kappa score for each case vs. control classifier. Metrics were calculated from the predictions on each test set in five-fold cross-validation. Datasets are ordered as in Figure 1. ART = arthritis, ASD = autism spectrum disorder, CDI = *Clostridium difficile* infection, CRC = colorectal cancer, EDD = enteric diarrheal disease, HIV = human immunodeficient virus, IBD = inflammatory bowel disease, LIV = liver disease, NASH = non-alcoholic steatohepatitis, nonCDI = non-*Clostridium difficile* infection, OB = obesity, PAR = Parkinson's disease, T1D = type I diabetes.

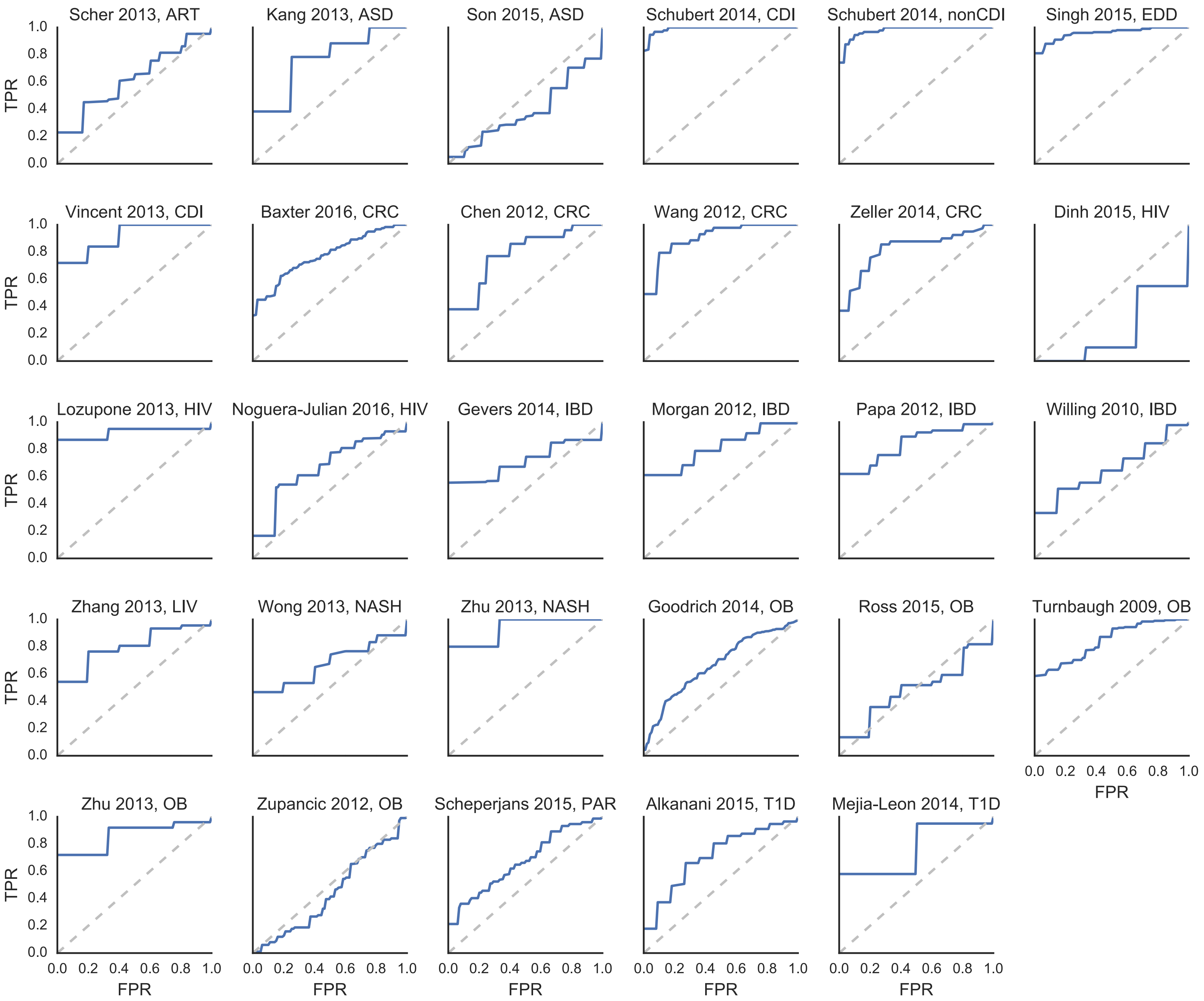

Supplementary Figure 1: ROC curves for each of the classifiers in Figure 1. Datasets are ordered alphabetically by disease and within disease by first author. FPR = false positive rate, TPR = true positive rate. ART = arthritis, ASD = autism spectrum disorder, CDI = *Clostridium difficile* infection, CRC = colorectal cancer, EDD = enteric diarrheal disease, HIV = human immunodeficient virus, IBD = inflammatory bowel disease, LIV = liver disease, NASH = non-alcoholic steatohepatitis, nonCDI = non-*Clostridium difficile* infection, OB = obesity, PAR = Parkinson's disease, T1D = type I diabetes.

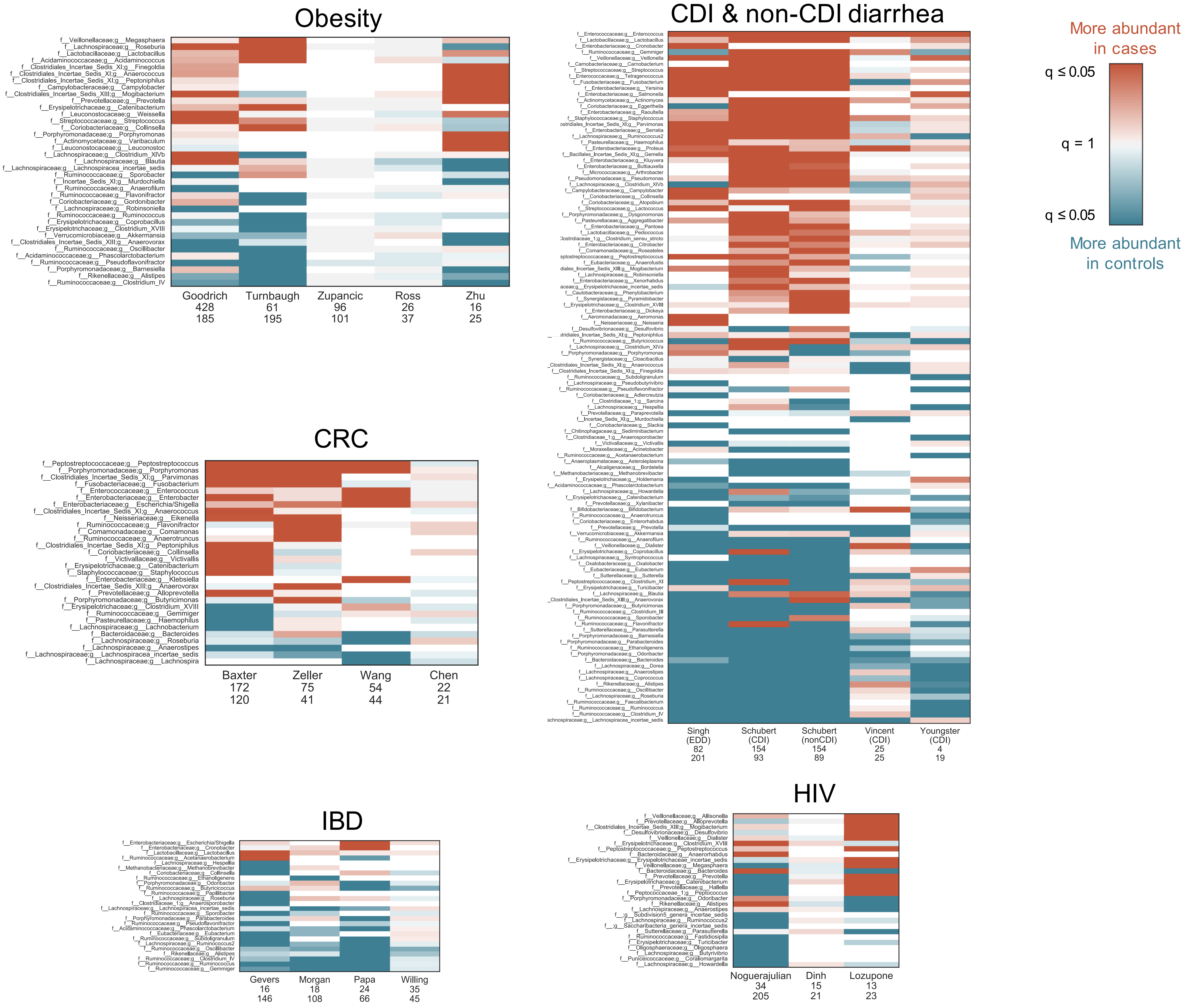

Supplementary Figure 2: Same heatmaps as in Figure 2, with rows labeled by family and genus taxonomy. Heatmaps show log10(q-values) for each disease (Kruskal-Wallis (KW) test, Benjamini-Hochberg FDR correction). Rows include all genera which were significant in at least one dataset within each disease, columns are datasets. Q-values are colored by direction of the effect, where red indicates higher mean abundance in disease patients and blue indicates higher mean abundance in controls. Opacity ranges from q = 0.05 to 1, where q values less than 0.05 are the most opaque and q values close to 1 are gray. White indicates that the genus was not present in that dataset. Within each heatmap, rows are ordered from most disease-associated (top) to most health-associated (bottom) (i.e. by the sum across rows of the log10(q-values), signed according to directionality of the effect).

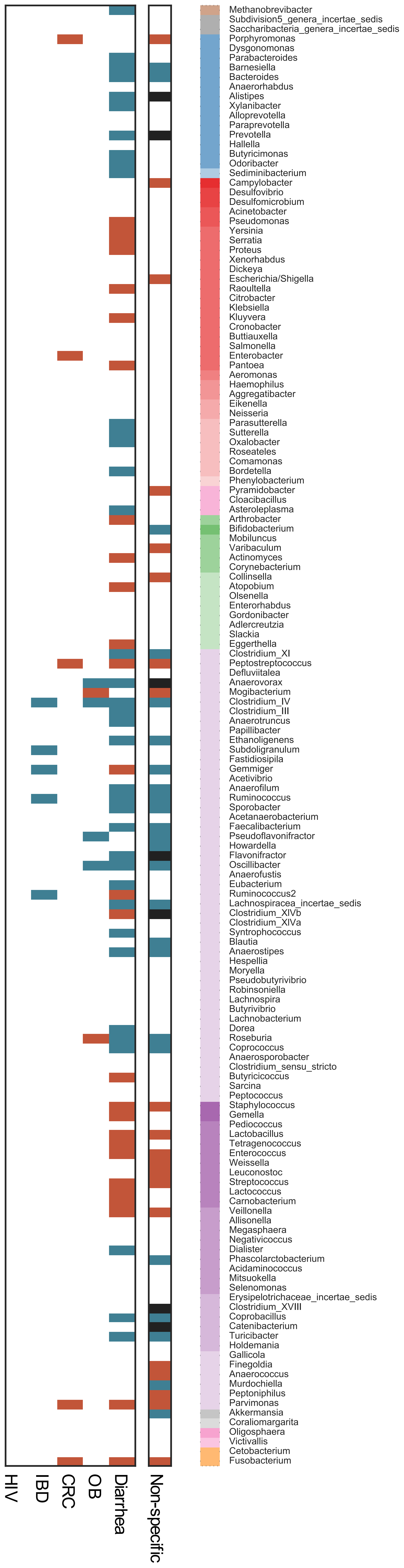

Supplementary Figure 3: Panel A from Figure 3, with genus labels. Non-specific and disease-associated genera. Genera are in rows, arranged phylogenetically according to a PhyloT tree built from genus-level NCBI IDs (`http://phylot.biobyte.de`). Non-specific genera are associated with health (or disease) in at least two different *diseases* ($q < 0.05$, Kruskal-Wallis (KW) test, Benjamini-Hochberg FDR correction). Disease-specific genera are significant in the same direction in at least two *studies* of the same disease ($q < 0.05$, FDR KW test). As in Figure 2, blue indicates higher mean abundance in controls and red indicates higher mean abundance in patients. Black bars indicate mixed genera which were associated with health in two diseases and also associated with disease in two diseases. Disease-specific genera are shown for diseases with at least 3 studies.

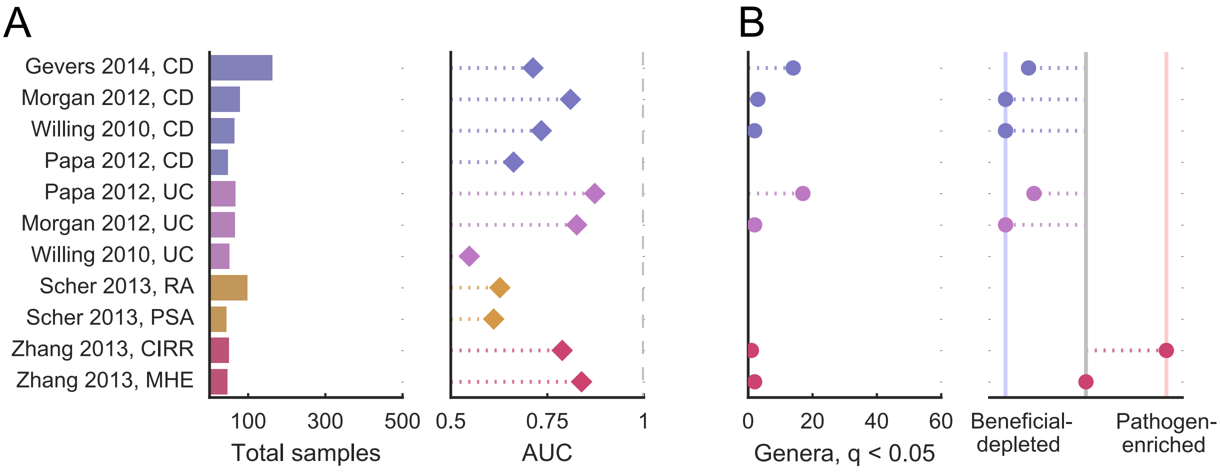

Supplementary Figure 4: Same analysis as in Figure 1 for stratified patient groups. (A) Left: Total sample size for each comparison. Right: Area under the ROC curve (AUC) for genus-level random forest classifiers. (B) Left: Number of genera with $q < 0.05$ (Kruskal-Wallis (KW) test, Benjamini-Hochberg FDR correction) for each type of patient group comparison. Right: Direction of microbiome shift,i.e. the percent of total associated genera which were enriched in diseased patients. In comparisons on the leftmost blue line, 100% of associated ($q < 0.05$, FDR KW test) genera are health-associated (i.e. depleted in patients relative to controls). In comparisons on the rightmost red line, 100% of associated ($q < 0.05$, FDR KW test) genera are disease-associated (i.e. enriched in patients relative to controls).

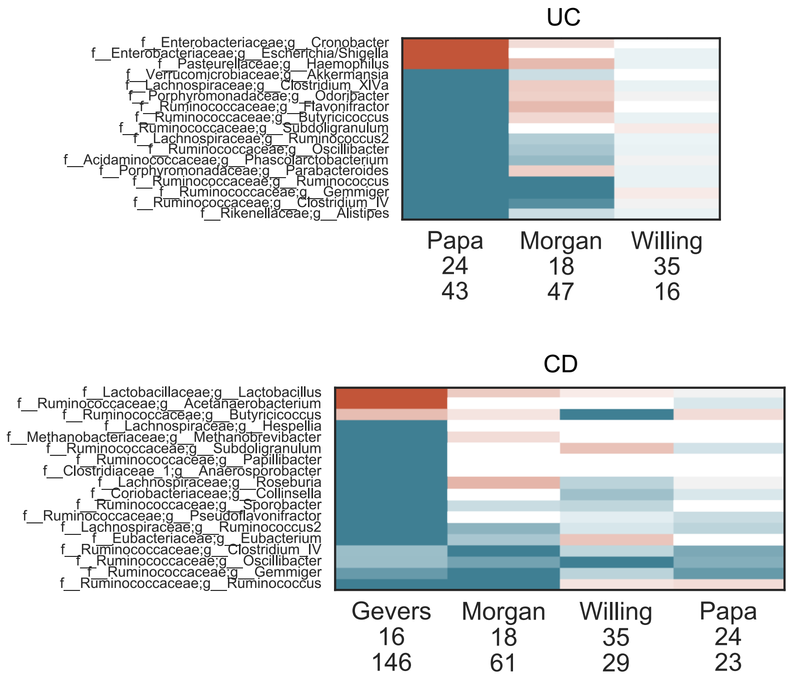

Supplementary Figure 5: Same results as presented in Figure 2 for ulcerative colitis (UC) and Crohn's disease (CD) IBD patients separately. Heatmaps show log10(q-values) for each comparison, with studies in columns and genera in rows (Kruskal-Wallis (KW) test, Benjamini-Hochberg FDR correction). Q-values are colored by direction of the effect, where red indicates higher mean abundance in disease patients and blue indicates higher mean abundance in controls. Opacity ranges from q = 0.05 to 1, where q values less than 0.05 are the most opaque and q values close to 1 are gray. White indicates that the genus was not present in that dataset. Within each heatmap, rows are ordered from most disease-associated (top) to most health-associated (bottom) (i.e. by the sum across rows of the log10(q-values), signed according to directionality of the effect).

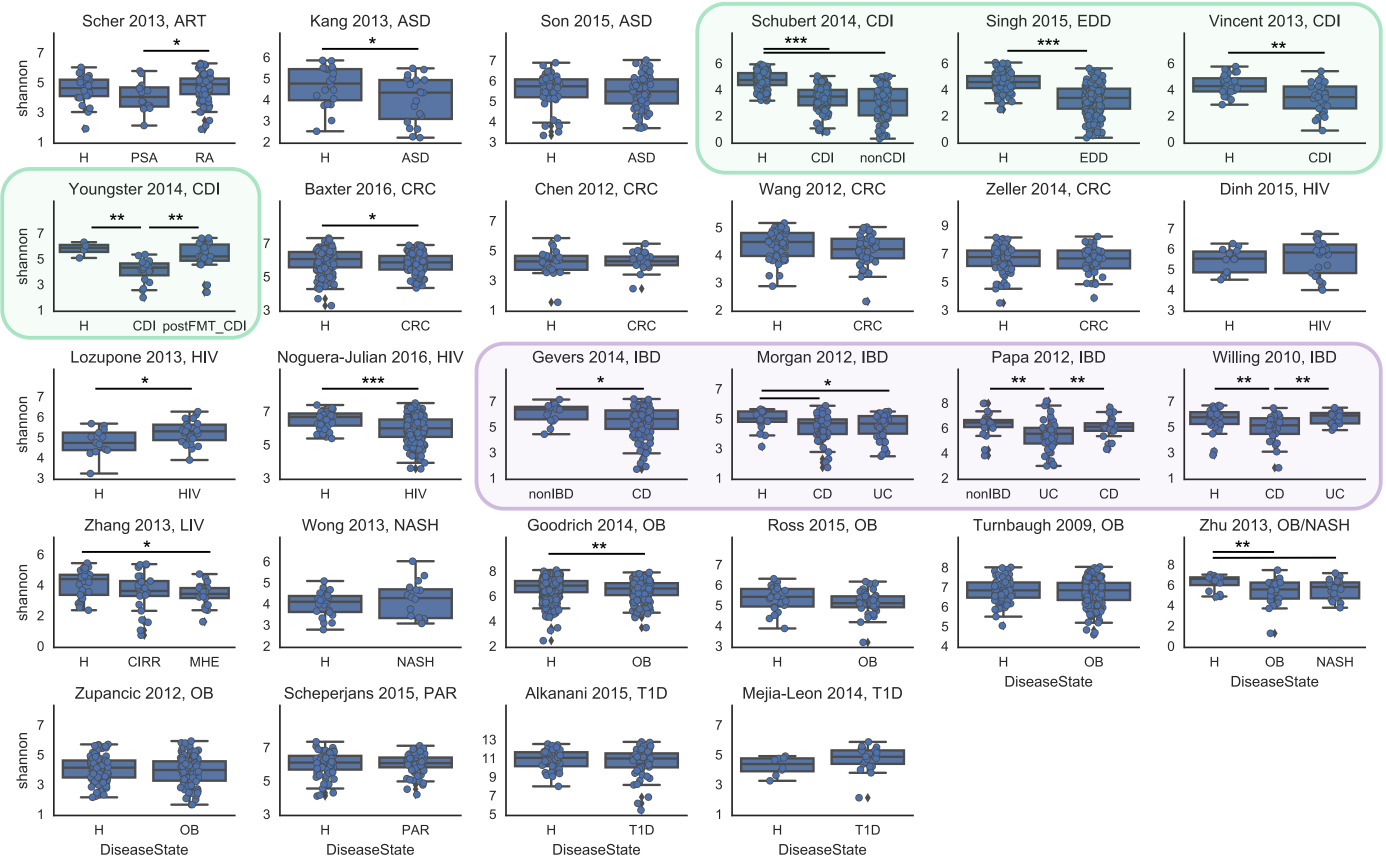

Supplementary Figure 6: Reduction in alpha diversity is not a reliable indicator of "dysbiosis." Shannon alpha diversity index across all patient groups in all studies, calculated on OTUs (i.e. not collapsed to genus level, and including unannotated OTUs). Diarrheal patients consistently have lower alpha diversity than non-diarrheal controls (green box). Crohn's disease (CD) patients also show a slight reduction of alpha diversity relative to controls in three out of four IBD studies and ulcerative colitis (UC) patients in two studies (purple box). Obese patients have inconsistent and small reductions in alpha diversity, consistent with a previous meta-analysis.[20] $* : 0.01 < p < 0.05, ** : 10^{-4} < p < 0.01, *** : p < 10^{-4}$. P values are calculated from a two-sided T-test (using `scipy.stats.ttest_ind`) and are not corrected for multiple tests. Note that the datasets with multiple case groups (Zhu et al. (OB/NASH, 2013) and Schubert et al. (CDI/non-CDI, 2014)) are presented only once in this plot. ART = arthritis, ASD = austism spectrum disorder, CD = Crohn's disease, CDI = *Clostridium difficile* infection, CIRR = liver cirrhosis, CRC = colorectal cancer, EDD = enteric diarrheal disease, H = healthy, HIV = human immunodeficiency virus, LIV = liver diseases, MHE = minimal hepatic encephalopathy, NASH = non-alcoholic steatohepatitis, OB = obesity, PAR = Parkinson's disease, PSA = psoriatic arthritis, RA = rheumatoid arthritis, T1D = type I diabetes, UC = ulcerative colitis. nonCDI controls are patients with diarrhea who tested negative for *C. difficile* infection. nonIBD controls are patients with gastrointestinal symptoms but no intestinal inflammation.

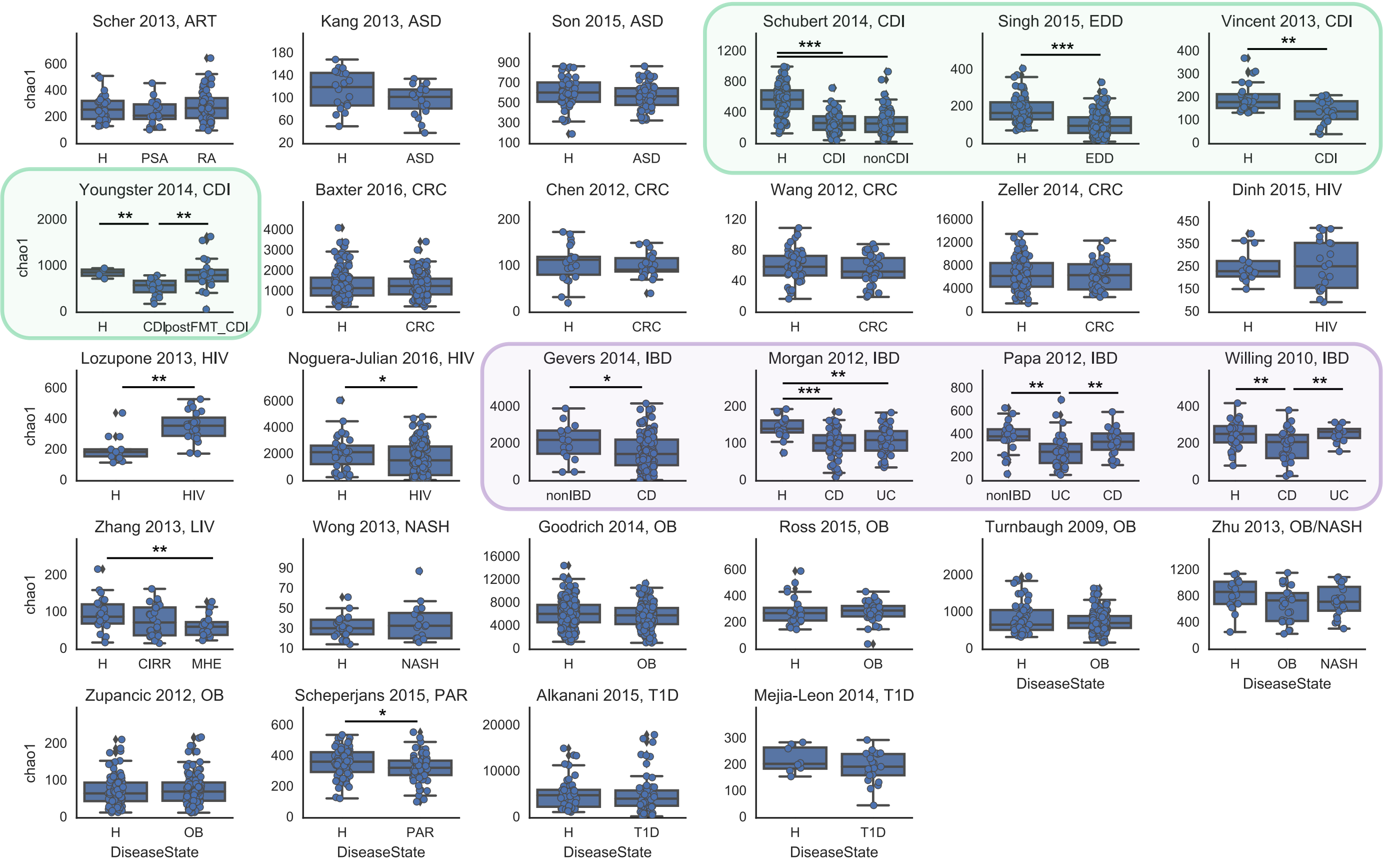

Supplementary Figure 7: Chao1 alpha diversity across all patient groups in all studies, calculated on OTUs (i.e. not collapsed to genus level, and including unannotated OTUs). $* : 0.01 < p < 0.05, ** : 10^{-4} < p < 0.01, *** : p < 10^{-4}$. P values are calculated from a two-sided T-test (using `scipy.stats.ttest_ind`) and are not corrected for multiple tests. Note that the datasets with multiple case groups (Zhu et al. (OB/NASH, 2013) and Schubert et al. (CDI/non-CDI, 2014)) are presented only once in this plot. ART = arthritis, ASD = austism spectrum disorder, CD = Crohn's disease, CDI = *Clostridium difficile* infection, CIRR = liver cirrhosis, CRC = colorectal cancer, EDD = enteric diarrheal disease, H = healthy, HIV = human immunodeficiency virus, LIV = liver diseases, MHE = minimal hepatic encephalopathy, NASH = non-alcoholic steatohepatitis, OB = obesity, PAR = Parkinson's disease, PSA = psoriatic arthritis, RA = rheumatoid arthritis, T1D = type I diabetes, UC = ulcerative colitis. nonCDI controls are patients with diarrhea who tested negative for *C. difficile* infection. nonIBD controls are patients with gastrointestinal symptoms but no intestinal inflammation.

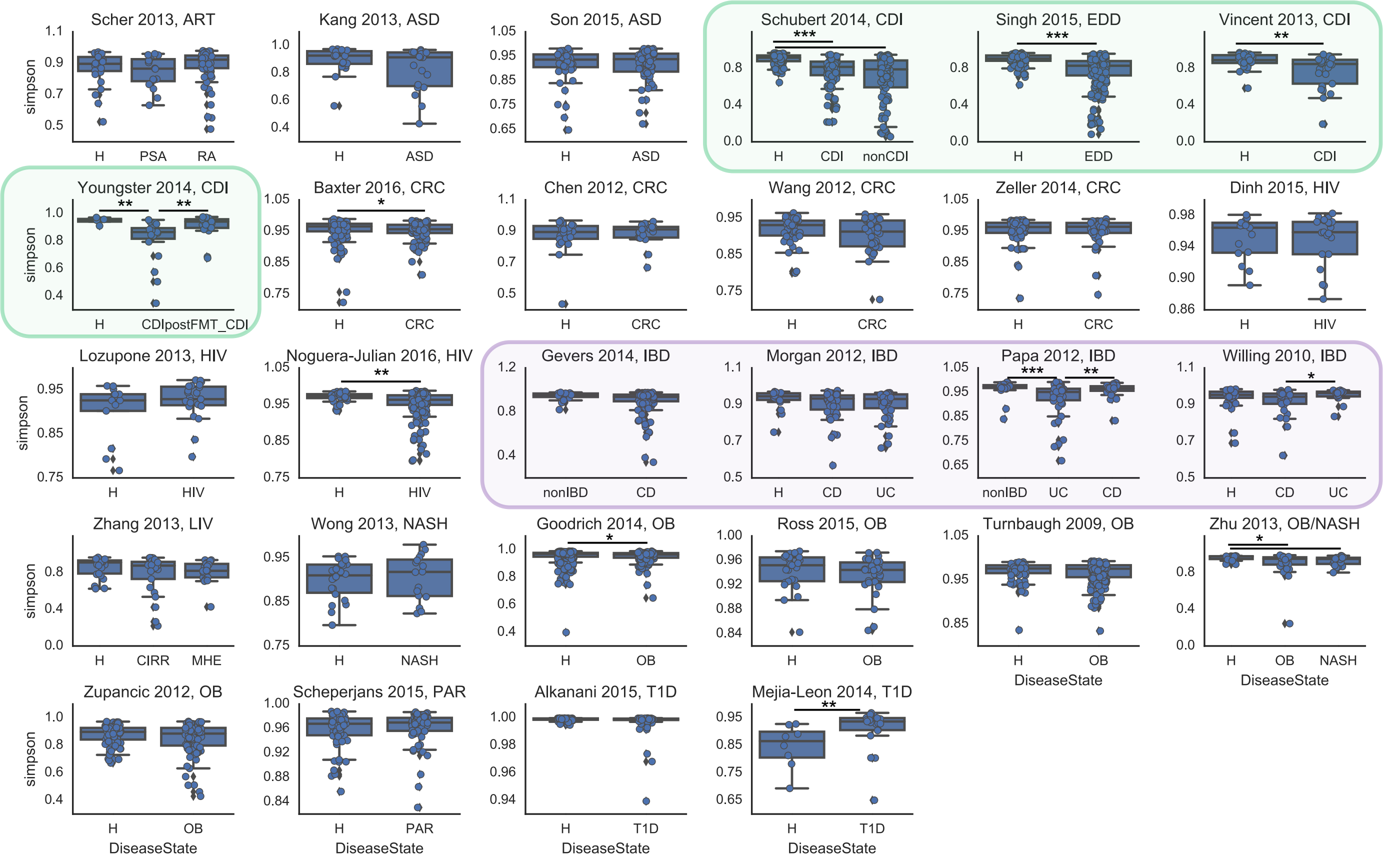

Supplementary Figure 8: Simpson alpha diversity across all patient groups in all studies, calculated on OTUs (i.e. not collapsed to genus level, and including unannotated OTUs). $* : 0.01 < p < 0.05, ** : 10^{-4} < p < 0.01, *** : p < 10^{-4}$. P values are calculated from a two-sided T-test (using `scipy.stats.ttest_ind`) and are not corrected for multiple tests. Note that the datasets with multiple case groups (Zhu et al. (OB/NASH, 2013) and Schubert et al. (CDI/non-CDI, 2014)) are presented only once in this plot. ART = arthritis, ASD = austism spectrum disorder, CD = Crohn's disease, CDI = *Clostridium difficile* infection, CIRR = liver cirrhosis, CRC = colorectal cancer, EDD = enteric diarrheal disease, H = healthy, HIV = human immunodeficiency virus, LIV = liver diseases, MHE = minimal hepatic encephalopathy, NASH = non-alcoholic steatohepatitis, OB = obesity, PAR = Parkinson's disease, PSA = psoriatic arthritis, RA = rheumatoid arthritis, T1D = type I diabetes, UC = ulcerative colitis. nonCDI controls are patients with diarrhea who tested negative for *C. difficile* infection. nonIBD controls are patients with gastrointestinal symptoms but no intestinal inflammation.

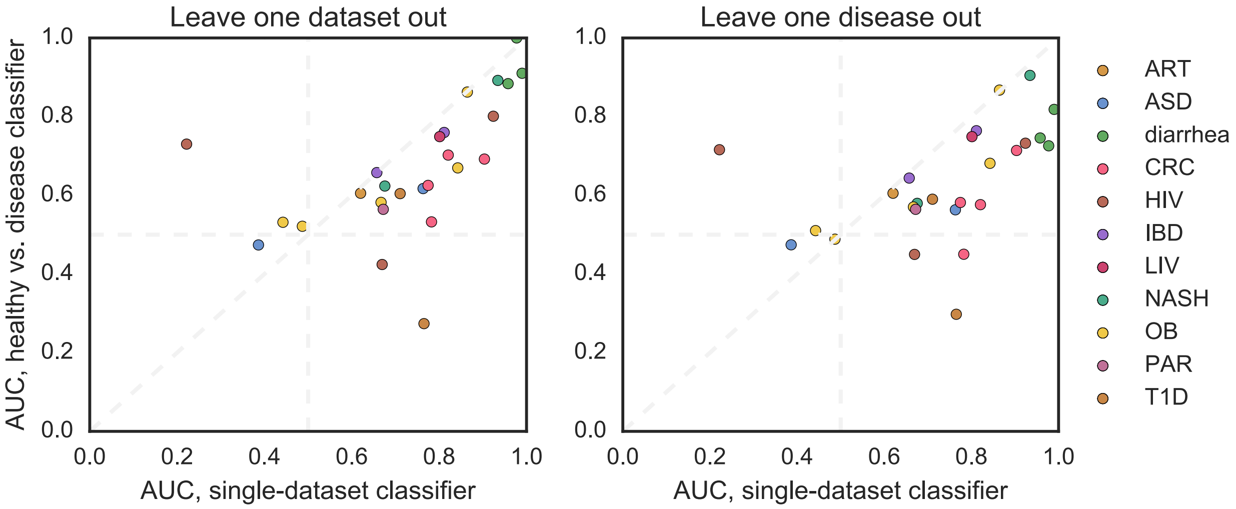

Supplementary Figure 9: *Both x-axes*: the area under the ROC curve (AUC) from each datasets single classifier. Left: leave-one-dataset-out classifier. *y-axis*: the AUC of a classifier trained on all other datasets to distinguish healthy from unhealthy patients, tested on the left out dataset. Right: leave-one-disease-out classifier. *y-axis*: AUC from a classifier trained to distinguish healthy from unhealthy patients on all datasets except those of the tested disease. AUCs for each dataset were built from the classification probabilities on each test sample.

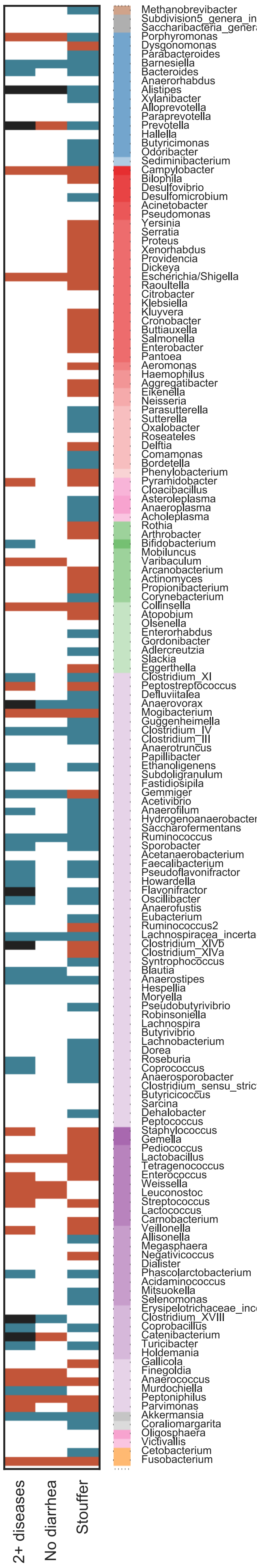

Supplementary Figure 10: The majority of non-specific microbes are robust to the exclusion of diarrhea datasets from consideration. The right-most bar shows order-level phylogeny, colored as in Figure 3A of the main paper. The left bar of the heatmap shows the original non-specific microbes, including all datasets. The middle bar shows the re-defined non-specific responders after excluding all diarrhea datasets. The right bar of the heatmap shows the non-specific microbes defined using Stouffers method, combining one-tailed q-values across datasets and weighting by the square root of sample size (Stouffer combined $q < 0.05$).

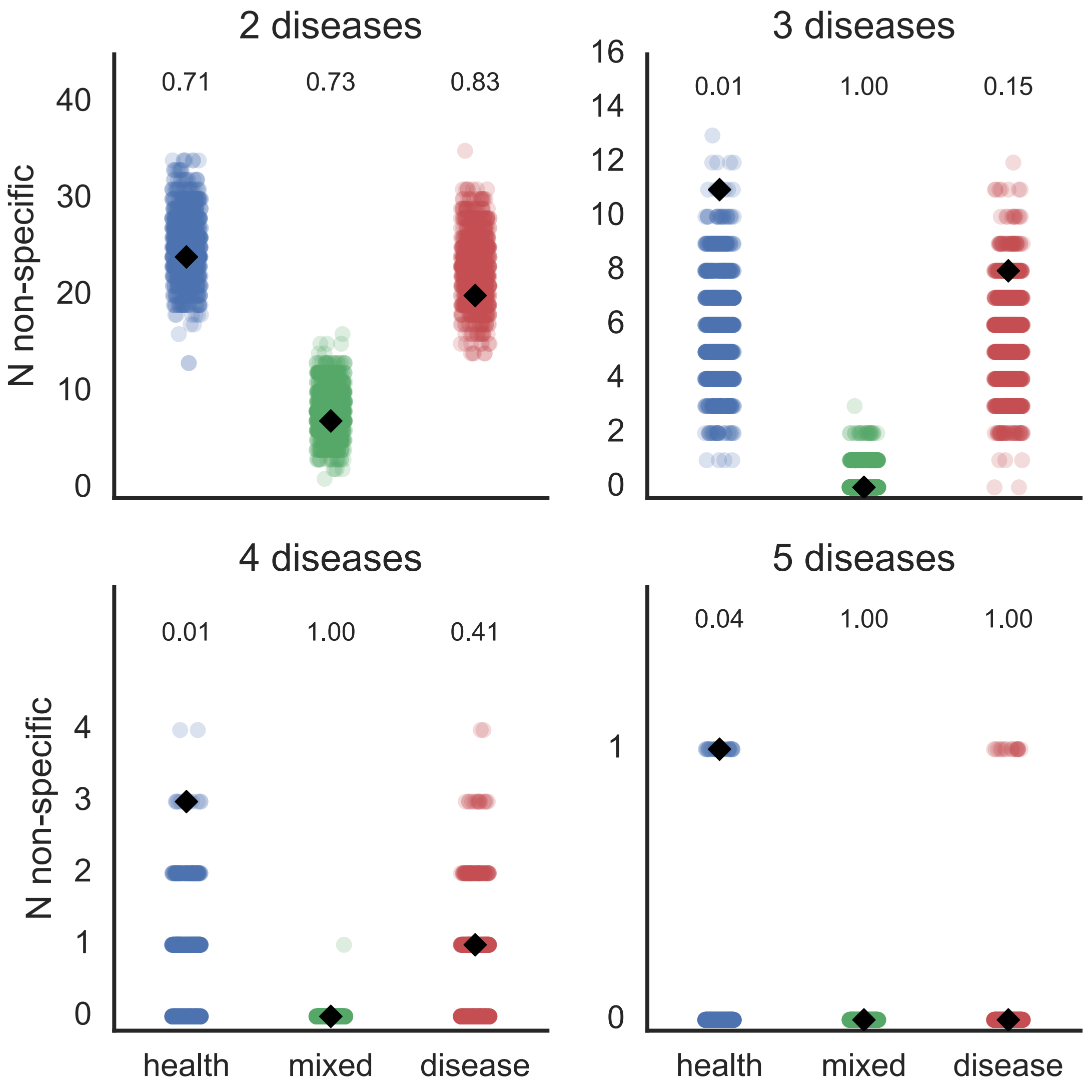

Supplementary Figure 11: Empirical null distribution of the number of non-specific responders (colored points, x-axis indicates directionality of response), overlayed with the actual observed number of non-specific responders (black diamonds) for different defining heuristics (axis titles, i.e. "3 diseases" means that a genus needed to be significant ($q < 0.05$, Kruskal-Wallis (KW) test, Benjamini-Hochberg FDR correction) in three diseases in the same direction to be considered a non-specific responder). Empirical one-tailed p-values are printed above each distribution.

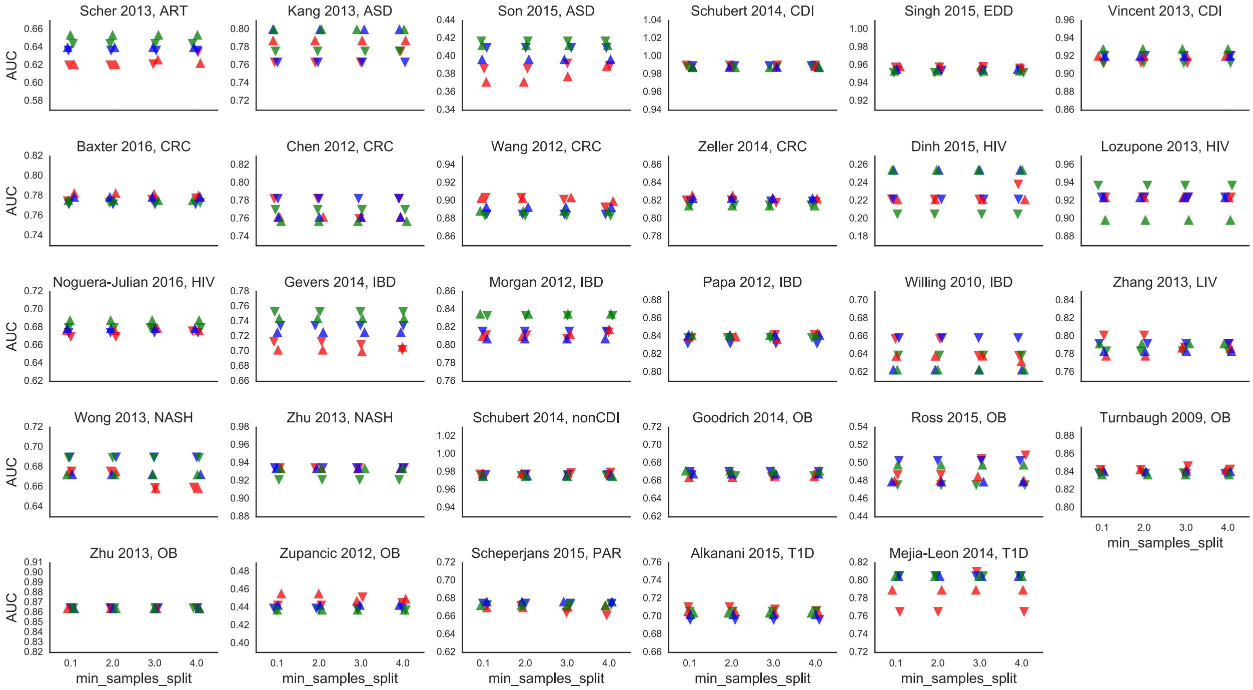

Supplementary Figure 12: Varying Random Forest parameters does not significantly affect area under the ROC curve in classifying cases from controls (Gini criteria). Random Forest classifiers built by using the Gini impurity ("gini") split criteria ("scikit-learn RandomForestClassifier"). Upward-pointing triangles are classifiers built with 10000 estimators; downward-pointing triangles are built with 1000 estimators. Colors indicate the value of `min_samples_leaf` (the minimum number of samples required to be at a leaf node): red = 1, blue = 2, green = 3. X-axes are the value of `min_samples_split` (the minimum number of samples required to split an internal node).[33] All Random Forests were built using the random state seed 12345.

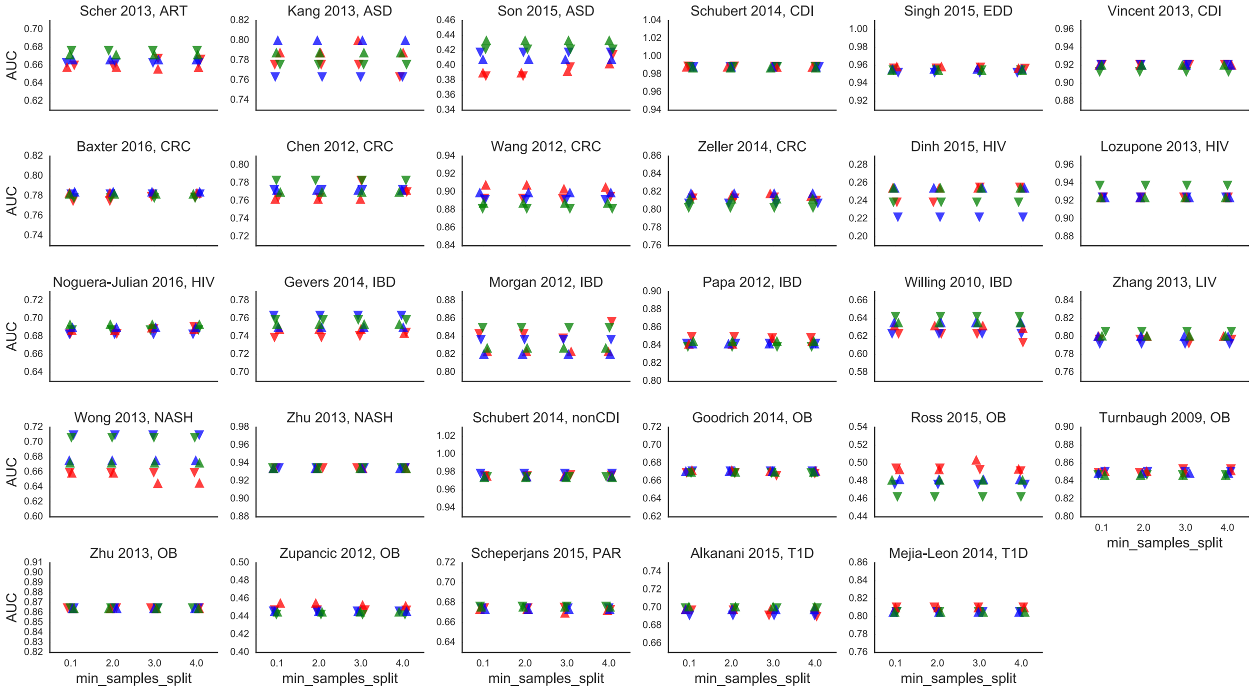

Supplementary Figure 13: Varying Random Forest parameters does not significantly affect area under the ROC curve in classifying cases from controls (entropy criteria). Random Forest classifiers built by using the entropy ("entropy") split criteria ("scikit-learn RandomForestClassifier"). Upward-pointing triangles are classifiers built with 10000 estimators; downward-pointing triangles are built with 1000 estimators. Colors indicate the value of `min_samples_leaf` (the minimum number of samples required to be at a leaf node): red = 1, blue = 2, green = 3. X-axes are the value of `min_samples_split` (the minimum number of samples required to split an internal node).[33] All Random Forests were built using the random state seed 12345.

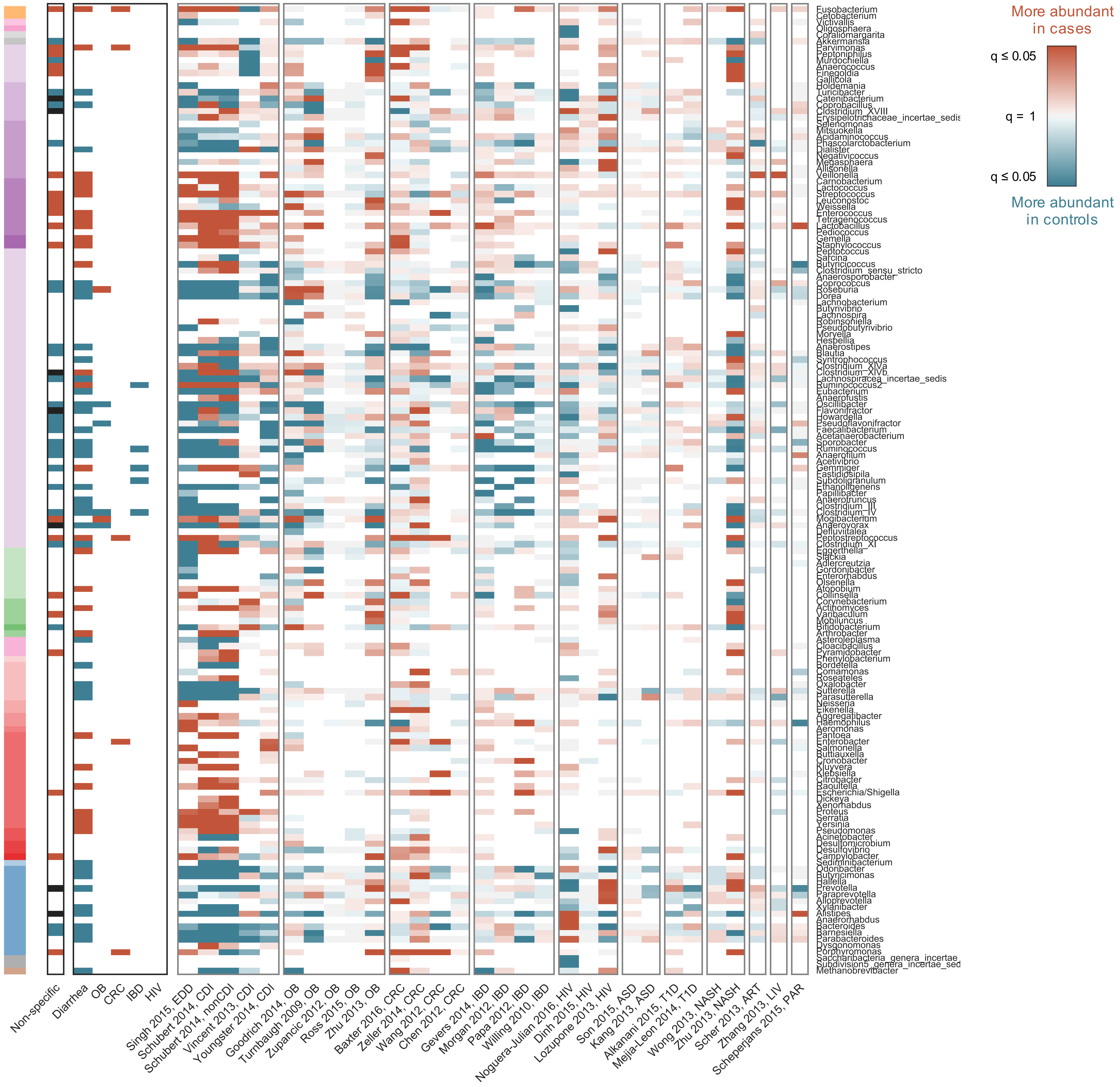

Supplementary Figure 14: Heatmap of log10(q values) for all genera which were significant ($q < 0.05$, Kruskal-Wallis (KW) test, Benjamini-Hochberg FDR correction) in at least one dataset, across all studies. Rows are genera, ordered phylogenetically (as in Figure 3A). Columns are datasets, grouped by disease and ordered according to total sample size (decreasing from left to right). The first and second heatmap panels from the left are the same as in Figure 3A. Q-values are colored according to directionality of the effect, where red indicates higher mean abundance in patients relative to controls and blue indicates higher mean abundance in controls. Opacity indicates significance and ranges from 0.05 to 1, where q-values less than 0.05 are the darkest colors and q-values close to 1 are gray. White indicates that the genus was not present in that dataset. ART = arthritis, ASD = autism spectrum disorder, CDI = *Clostridium difficile* infection, CRC = colorectal cancer, EDD = enteric diarrheal disease, HIV = human immunodeficient virus, IBD = inflammatory bowel disease, LIV = liver disease, NASH = non-alcoholic steatohepatitis, nonCDI = non-*Clostridium difficile* infection, OB = obesity, PAR = Parkinson's disease, T1D = type I diabetes.

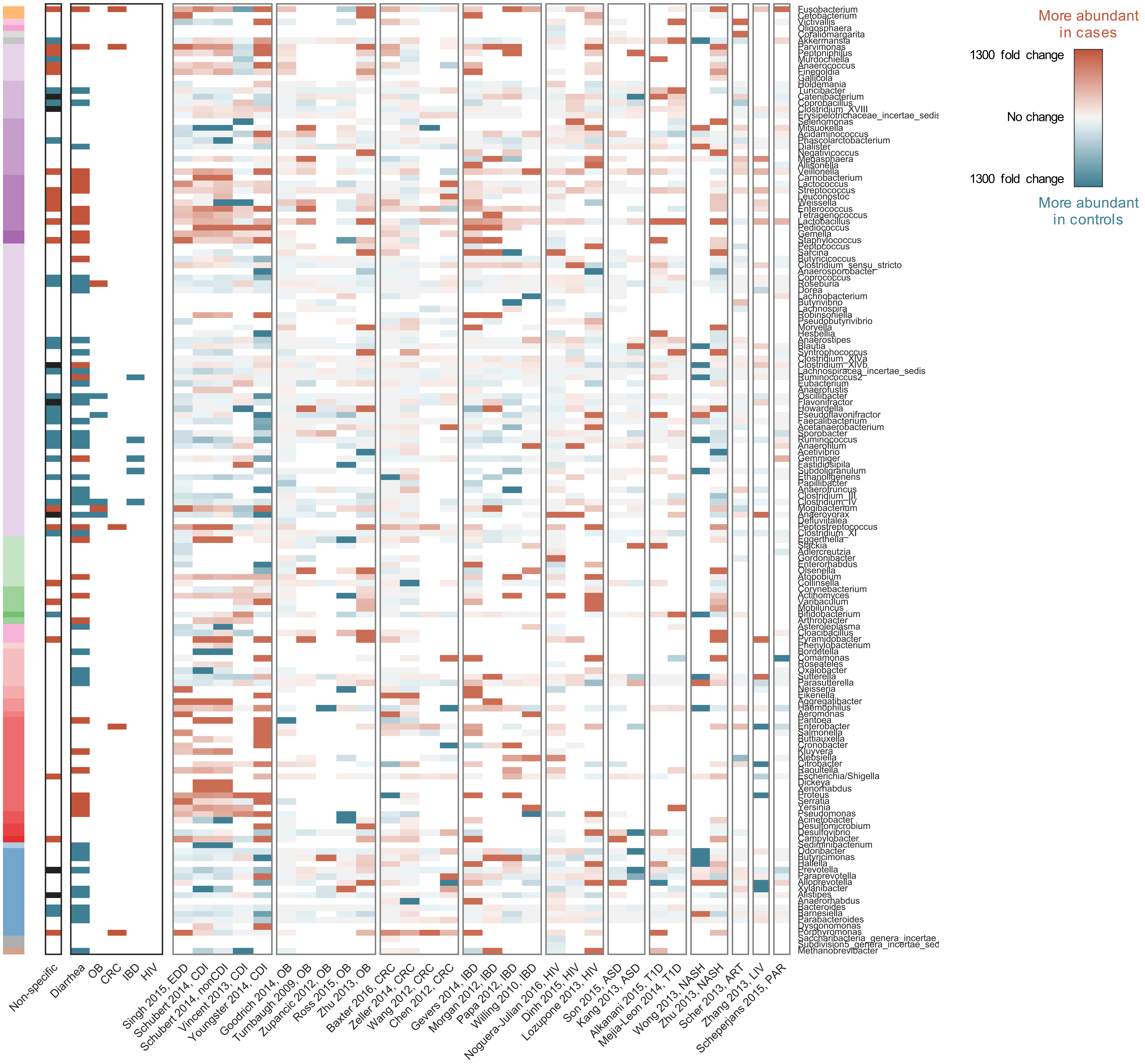

Supplementary Figure 15: Heatmap of log-fold change between cases and controls (i.e. $log_2(\frac{\text{mean abundance in cases}}{\text{mean abundance in controls}})$ for all genera which were significant ($q < 0.05$) in at least one dataset, across all studies. Rows are genera, ordered phylogenetically (as in Figure 3A). Columns are datasets, grouped by disease and ordered according to total sample size (decreasing from left to right). The first and second heatmap panels from the left are the same as in Figure 3A. Values are colored according to directionality of the effect, where red indicates higher mean abundance in patients relative to controls and blue indicates higher mean abundance in controls. Opacity indicates fold change and ranges from 1300 to 0, where fold changes greater than 1300 are the darkest colors and fold changes close to 0 are gray. White indicates that the genus was not present in that dataset. ART = arthritis, ASD = autism spectrum disorder, CDI = *Clostridium difficile* infection, CRC = colorectal cancer, EDD = enteric diarrheal disease, HIV = human immunodeficient virus, IBD = inflammatory bowel disease, LIV = liver disease, NASH = non-alcoholic steatohepatitis, nonCDI = non-*Clostridium difficile* infection, OB = obesity, PAR = Parkinson's disease, T1D = type I diabetes.

Supplementary References

[1] A.M. Schubert, M.A. Rogers, C. Ring, J. Mogle, J.P. Petrosino, V.B. Young, D.M. Aronoff, and P.D. Schloss. Microbiome data distinguish patients with clostridium difficile infection and non-c. difficile-associated diarrhea from healthy controls. *mBio*, 5(3):e01021–14–e01021–14, 2014.

[2] C. Vincent, D.A. Stephens, V.G. Loo, T.J. Edens, M.A. Behr, K. Dewar, and A.R. Manges. Reductions in intestinal clostridiales precede the development of nosocomial clostridium difficile infection. *Microbiome*, 1(1):18, 2013.

[3] I. Youngster, J. Sauk, C. Pindar, R.G. Wilson, J.L. Kaplan, M.B. Smith, E.J. Alm, D. Gevers, G.H. Russell, and E.L. Hohmann. Fecal microbiota transplant for relapsing clostridium difficile infection using a frozen inoculum from unrelated donors: A randomized, open-label, controlled pilot study. *Clinical Infectious Diseases*, 58(11):1515–1522, 2014.

[4] P. Singh, T.K. Teal, T.L. Marsh, J.M. Tiedje, R. Mosci, K. Jernigan, A. Zell, D.W. Newton, H. Salimnia, P. Lephart, D. Sundin, W. Khalife, R.A. Britton, J.T. Rudrik, and S.D. Manning. Intestinal microbial communities associated with acute enteric infections and disease recovery. *Microbiome*, 3(1), sep 2015.

[5] Gregory P Donaldson, S Melanie Lee, and Sarkis K Mazmanian. Gut biogeography of the bacterial microbiota. *Nature Reviews Microbiology*, 14(1):20–32, 2016.

[6] N.T. Baxter, M.T. Ruffin, M.A. Rogers, and P.D. Schloss. Microbiota-based model improves the sensitivity of fecal immunochemical test for detecting colonic lesions. *Genome Medicine*, 8(1), 2016.

[7] Caitlin A Brennan and Wendy S Garrett. The gut microbiome, inflammation, and colorectal cancer. *Annual Review of Microbiology*, 70(1), 2016.

[8] G. Zeller, J. Tap, A.Y. Voigt, S. Sunagawa, J.R. Kultima, P.I. Costea, A. Amiot, J. Bohm, F. Brunetti, N. Habermann, R. Hercog, M. Koch, A. Luciani, D.R. Mende, M.A. Schneider, P. Schrotz-King, C. Tournigand, J.T. Nhieu, T. Yamada, J. Zimmermann, V. Benes, M. Kloor, C.M. Ulrich, M. von Knebel Doeberitz, I. Sobhani,

and P. Bork. Potential of fecal microbiota for early-stage detection of colorectal cancer. *Molecular Systems Biology*, 10(11):766–766, 2014.

[9] T. Wang, G. Cai, Y. Qiu, N. Fei, M. Zhang, X. Pang, W. Jia, S. Cai, and L. Zhao. Structural segregation of gut microbiota between colorectal cancer patients and healthy volunteers. *The ISME Journal*, 6(2):320–329, 2011.

[10] W. Chen, F. Liu, Z. Ling, X. Tong, and C. Xiang. Human intestinal lumen and mucosa-associated microbiota in patients with colorectal cancer. *PLoS ONE*, 7(6):e39743, 2012.

[11] D. Gevers, S. Kugathasan, L.A. Denson, Y. Vázquez-Baeza, W. Van Treuren, B. Ren, E. Schwager, D. Knights, S. Song, M. Yassour, X.C. Morgan, A.D. Kostic, C. Luo, A. González, D. McDonald, Y. Haberman, T. Walters, S. Baker, J. Rosh, M. Stephens, M. Heyman, J. Markowitz, R. Baldassano, A. Griffiths, F. Sylvester, D. Mack, S. Kim, W. Crandall, J. Hyams, C. Huttenhower, R. Knight, and R. Xavier. The treatment-naive microbiome in new-onset crohn's disease. *Cell Host & Microbe*, 15(3):382–392, mar 2014.

[12] X.C. Morgan, T.L. Tickle, H. Sokol, D. Gevers, K.L. Devaney, D.V. Ward, J.A. Reyes, S.A. Shah, N. LeLeiko, S.B. Snapper, A. Bousvaros, J. Korzenik, B.E. Sands, R.J. Xavier, and C. Huttenhower. Dysfunction of the intestinal microbiome in inflammatory bowel disease and treatment. *Genome Biol*, 13(9):R79, 2012.

[13] E. Papa, M. Docktor, C. Smillie, S. Weber, S.P. Preheim, D. Gevers, G. Giannoukos, D. Ciulla, D. Tabbaa, J. Ingram, D.B. Schauer, D.V. Ward, J.R. Korzenik, R.J. Xavier, A. Bousvaros, and E.J. Alm. Non-invasive mapping of the gastrointestinal microbiota identifies children with inflammatory bowel disease. *PLoS ONE*, 7(6):e39242, 2012.

[14] B.P. Willing, J. Dicksved, J. Halfvarson, A.F. Andersson, M. Lucio, Z. Zheng, G. J"arnerot, C. Tysk, J.K. Jansson, and L. Engstrand. A pyrosequencing study in twins shows that gastrointestinal microbial profiles vary with inflammatory bowel disease phenotypes. *Gastroenterology*, 139(6):1844–1854.e1, dec 2010.

[15] J.K. Goodrich, J.L. Waters, A.C. Poole, J.L. Sutter, O. Koren, R. Blekhman, M. Beaumont, W. Van Treuren, R. Knight, J.T. Bell, T.D. Spector, A.G. Clark,

and R.E. Ley. Human genetics shape the gut microbiome. *Cell*, 159(4):789–799, nov 2014.

[16] M.L. Zupancic, B.L. Cantarel, Z. Liu, E.F. Drabek, K.A. Ryan, S. Cirimotich, C. Jones, R. Knight, W.A. Walters, D. Knights, E.F. Mongodin, R.B. Horenstein, B.D. Mitchell, N. Steinle, S. Snitker, A.R. Shuldiner, and C.M. Fraser. Analysis of the gut microbiota in the old order Amish and its relation to the metabolic syndrome. *PloS one*, 7(8):e43052, 2012.

[17] P.J. Turnbaugh, M. Hamady, T. Yatsunenko, B.L. Cantarel, A. Duncan, R.E. Ley, M.L. Sogin, W.J. Jones, B.A. Roe, J.P. Affourtit, M. Egholm, B. Henrissat, A.C. Heath, R. Knight, and J.I. Gordon. A core gut microbiome in obese and lean twins. *Nature*, 457(7228):480–484, 2008.

[18] M.C. Ross, D.M. Muzny, J.B. McCormick, R.A. Gibbs, S.P. Fisher-Hoch, and J.F. Petrosino. 16s gut community of the cameron county hispanic cohort. *Microbiome*, 3(1):7, 2015.

[19] L. Zhu, S.S. Baker, C. Gill, W. Liu, R. Alkhouri, R.D. Baker, and S.R. Gill. Characterization of gut microbiomes in nonalcoholic steatohepatitis (NASH) patients: a connection between endogenous alcohol and NASH. *Hepatology*, 57(2):601–609, 2013.

[20] Marc A. Sze and Patrick D. Schloss. Looking for a signal in the noise: Revisiting obesity and the microbiome. *mBio*, 7(4), 2016.

[21] D.M. Dinh, G.E. Volpe, C. Duffalo, S. Bhalchandra, A.K. Tai, A.V. Kane, C.A. Wanke, and H.D. Ward. Intestinal microbiota, microbial translocation, and systemic inflammation in chronic HIV infection. *Journal of Infectious Diseases*, 211(1):19–27, 2014.

[22] Catherine A Lozupone, Marcella Li, Thomas B Campbell, Sonia C Flores, Derek Linderman, Matthew J Gebert, Rob Knight, Andrew P Fontenot, and Brent E Palmer. Alterations in the gut microbiota associated with hiv-1 infection. *Cell host & microbe*, 14(3):329–339, 2013.

[23] D.W. Kang, J.G. Park, Z.E. Ilhan, G. Wallstrom, J. LaBaer, J.B. Adams, and R. Krajmalnik-Brown. Reduced incidence of Prevotella and other fermenters in intestinal microflora of autistic children. *PloS one*, 8(7):e68322, 2013.

[24] J. Son, L.J. Zheng, L.M. Rowehl, X. Tian, Y. Zhang, W. Zhu, L. Litcher-Kelly, K.D. Gadow, G. Gathungu, C.E. Robertson, D. Ir, D.N. Frank, and E. Li. Comparison of fecal microbiota in children with autism spectrum disorders and neurotypical siblings in the simons simplex collection. *PLOS ONE*, 10(10):e0137725, 2015.

[25] A.K. Alkanani, N. Hara, P.A. Gottlieb, D. Ir, C.E. Robertson, B.D. Wagner, D.N. Frank, and D. Zipris. Alterations in intestinal microbiota correlate with susceptibility to type 1 diabetes. *Diabetes*, 64(10):3510–3520, 2015.

[26] M.E. Mejía-León, J.F. Petrosino, N.J. Ajami, M.G. Domínguez-Bello, and A.M.C. de la Barca. Fecal microbiota imbalance in mexican children with type 1 diabetes. *Sci. Rep.*, 4, 2014.

[27] V.W. Wong, C. Tse, T.T. Lam, G.L. Wong, A.M. Chim, W.C. Chu, D.K. Yeung, P.T. Law, H. Kwan, J. Yu, J.J. Sung, and H.L. Chan. Molecular characterization of the fecal microbiota in patients with nonalcoholic steatohepatitis – a longitudinal study. *PLoS ONE*, 8(4):e62885, apr 2013.

[28] Z. Zhang, H. Zhai, J. Geng, R. Yu, H. Ren, H. Fan, and P. Shi. Large-scale survey of gut microbiota associated with MHE via 16s rRNA-based pyrosequencing. *Am J Gastroenterol*, 108(10):1601–1611, jul 2013.

[29] J.U. Scher, A. Sczesnak, R.S. Longman, N. Segata, C. Ubeda, C. Bielski, T. Rostron, V. Cerundolo, E.G. Pamer, S.B. Abramson, C. Huttenhower, and D.R. Littman. Expansion of intestinal prevotella copri correlates with enhanced susceptibility to arthritis. *eLife*, 2, 2013.

[30] F. Scheperjans, V. Aho, P.A.B. Pereira, K. Koskinen, L. Paulin, E. Pekkonen, E. Haapaniemi, S. Kaakkola, J. Eerola-Rautio, P. Pohja, E. Kinnunen, K. Murros, and P. Auvinen. Gut microbiota are related to parkinson's disease and clinical phenotype. *Movement Disorders*, 30(3):350–358, dec 2014.

[31] Samuel Andrew Stouffer, Edward A Suchman, Leland C De Vinney, Shirley A Star, and Robin M Williams. Studies in social psychology in world war II. vol. I: The american soldier: Adjustment during army life. 1951.

[32] Marc Noguera-Julian, Muntsa Rocafort, Yolanda Guillén, Javier Rivera, Maria Casadellà, Piotr Nowak, Falk Hildebrand, Georg Zeller, Mariona Parera, Rocío Bellido, et al. Gut microbiota linked to sexual preference and hiv infection. *EBioMedicine*, 5:135–146, 2016.

[33] F. Pedregosa, G. Varoquaux, A. Gramfort, V. Michel, B. Thirion, O. Grisel, M. Blondel, P. Prettenhofer, R. Weiss, V. Dubourg, J. Vanderplas, A. Passos, D. Cournapeau, M. Brucher, M. Perrot, and E. Duchesnay. Scikit-learn: Machine learning in Python. *Journal of Machine Learning Research*, 12:2825–2830, 2011.
